# Precise spike-timing information in the brainstem is well aligned with the needs of communication and the perception of environmental sounds

**DOI:** 10.1371/journal.pbio.3003213

**Published:** 2025-06-16

**Authors:** Chris Scholes, Stephen Coombes, Alan R. Palmer, William S. Rhode, Rob Mill, Christian J. Sumner

**Affiliations:** 1 School of Psychology, University of Nottingham, Nottingham, United Kingdom; 2 School of Mathematical Sciences, University of Nottingham, Nottingham, United Kingdom; 3 School of Medicine, University of Nottingham, Nottingham, United Kingdom; 4 Department of Physiology, University of Wisconsin, Madison, Wisconsin, United States of America; 5 MRC Institute of Hearing Research, Nottingham, United Kingdom,; 6 Department of Psychology, Nottingham Trent University, Nottingham, United Kingdom; University College London, UNITED KINGDOM OF GREAT BRITAIN AND NORTHERN IRELAND

## Abstract

The dynamic fluctuations in the amplitude of sound, known as sound envelopes, are ubiquitous in natural sounds and convey information critical for the recognition of speech, and of sounds generally. We are perceptually most sensitive to slow modulations which are most common. However, previous studies of envelope coding in the brainstem found an under-representation of these slow, low-frequency, modulations. Specifically, the synchronization of spike times to the envelope was enhanced in some neuron types, forming channels specialized for envelope processing but tuned to a restricted range of fast, high-frequency, envelopes (200–500 Hz). Here, we show using a historical dataset from cats that previous analyses, which made strong assumptions about the neural code, underestimated the encoding of low-frequency envelopes. While some neurons encode envelope better than others, most encode a wide range of envelope frequencies, and represent slower envelope fluctuations most accurately in their precise patterns of spike times. Identification of envelope frequency from spike-timing was linked to reliability, and to the way that dynamics of spiking interacted with the time-varying envelope. In some of the best-performing neurons, temporally complex “mode-locked” spike patterns served to enhance envelope coding. A second long-standing contradiction was that neural envelope coding is degraded at high sound levels, whilst the perception of envelope is robust at a wide range of sound levels. We find that spike-time encoding of envelope shape becomes level-robust for small populations of neurons. These findings argue against feature-specific coding of envelopes in the brainstem, and for a distributed population spike-time code for which synchrony to the envelope is an incomplete description. This code is accurate for slow fluctuations and robust across sound level. Thus, precise spike-timing information in the brainstem is after-all aligned with the needs of communication and the perception of environmental sounds.

## Abstract 2

Classical studies suggested that slow amplitude fluctuations in sounds critical for speech recognition are poorly represented in the brainstem. This paper shows that overlooked intricacies of spike timing represent these fluctuations, reconciling low-level neural processing with perception.

## Introduction

Our senses are confronted with dynamic physical signals that fluctuate over time. For sound, envelope fluctuations in different frequency bands carry most of the information required to recognize speech [1,2] and natural sounds [3]. In the early stages of the auditory system, envelopes of up to hundreds of cycles per second are represented in the precise timing of spikes [4–6]. As the auditory pathway is ascended, spike timing becomes restricted to encoding slower (a few 10 s of cycles per second) fluctuations [7–10], and firing rate codes [11] emerge, a transformation mirrored in other sensory systems [12]. Understanding how this chain of processing serves our perception of sound envelopes is key to understanding how we recognize speech and other complex sounds.

Here, we address two long-standing problems with our understanding of the low-level processing of envelopes in the brainstem. Neurons in the cochlear nucleus (CN) are the sole targets of the cochlear nerve fibers, and are the only neurons directly affected by cochlear hearing loss. Understanding how CN neurons represent and process the features in speech and environmental sounds may be critical in understanding the communication problems associated with poor hearing.

The temporal coding of sound envelope undergoes considerable transformation in the CN neurons which receive direct input from the cochlea. Cochlear nucleus neurons fire in synchrony with the envelope peaks, but different neuron types vary in their precision [5,13]. One sub-population of neurons appears specialized for envelope coding: regular-spiking “chopper” neurons [6,14] synchronize preferentially to specific modulation frequencies, and with a precision that exceeds their input nerve fibers [5,6]. Different chopper neurons most accurately synchronize to different envelope frequencies, ostensibly establishing channels which are specialized to encode different features of the envelope. This coding is transmitted directly to the midbrain (the inferior colliculus; IC), where among other transformations, a firing-rate tuning to modulation frequency emerges [15], though it remains a substantial challenge to understand how these transformations ultimately support robust coding of envelopes [16].

However, numerous discrepancies cast doubt on this account of envelope processing in the brainstem. Speech and natural sounds are dominated by low-frequency modulations down to a few cycles per second [3,17], whereas synchronized firing in chopper neurons is tuned to higher envelope modulation frequencies of 200–500 cycles per second. Such high-frequency tuning suggests chopper neurons are mainly suited to processing high-frequency modulations, which contribute weakly to our perception of pitch, but not the low-frequency modulations essential for communication. In addition, CN modulation tuning does not match the tuning observed in the IC [18], implying that modulation tuning at the next level is not inherited from the CN in any simple way. Other CN neuron types do show low-pass modulation tuning, but are overall less sensitive to envelope modulations [5,14], or are too broadly tuned to carrier frequency [19] to represent envelopes in the narrow frequency bands required to recognize speech [1,2]. In addition, in all CN neuron types, the precision of neural synchronization to the envelope decreases with increasing sound level, whereas human psychophysical performance is largely unaffected [20] or improves [21–23] with increases in level. Thus, although the importance of spike-timing is clear, exactly how this spike-timing supports the perception of ecologically important signals remains substantially unexplained. Inevitably, this gap in knowledge also limits our understanding of how cochlear hearing loss impacts on neural coding in the circuits which receive input directly from the cochlea.

We hypothesized that our understanding of the neural code for envelope in the CN may be incorrect, because key properties of the spike-timing have been overlooked. Neurophysiological studies of envelope coding often quantify the synchrony of spike timing to the modulation frequency: phase-locking [24]. This focus on phase-locking ignores any information that is not encoded as a preference to fire around a single phase of the envelope. Non-linear dynamics inherent in neurons [25,26] can interact with the dynamics of the input [27] resulting in neurons showing diverse, but precise and repeatable, spike-timing patterns to a given dynamic stimulus: “mode-locking”. In particular, chopper neurons are known to mode-lock to periodic envelope modulations [28], potentially conveying information not captured by phase-locking analysis. More generally, a great diversity of spike timing is observed in CN neurons [19,28–30]. Yet, the functional value of different modes of spike-timing is not known.

Here, we investigated what these spiking patterns mean for the coding of sound envelope. Using a spike-timing metric, which is sensitive to any reliable differences in spike timing, we find that the representation of modulations in a large dataset of CN neurons [6,31] remains precise at low modulation frequencies, and small ensembles of neurons provide robustness to sound level. Our results argue against the existence of channels which encode different envelope frequencies and in favor of a level-robust distributed temporal code. Such coding is more consistent with our perception and the preponderance of low-frequency envelopes in natural sounds.

## Results

We analyzed the responses of 336 neurons from previously published data [6,31], located throughout the CN, but mainly in the anteroventral and posteroventral parts of the CN of the cat (see Materials and methods), and with Characteristic Frequencies above 3 kHz where spike timing is determined by the envelope and not by temporal fine structure. Using standard classification based on the responses to unmodulated (pure) tones [32–35] the dataset was divided into 6 neuron response types (see Materials and methods): sustained chopper (ChS), transient chopper (ChT), primary-like (PL), primary-like notch (PLN), and a smaller sample of pause/build-up (PBU) from the dorsal region of CN, and onset (On) units.

**Fig 1:**
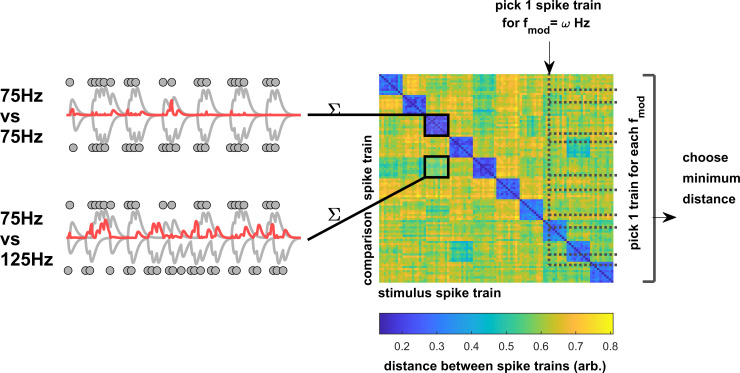
Quantifying differences between spike trains for a single neuron. Spike trains (times indicated by gray dots) are filtered by an alpha function (gray lines), and all pairs of spike trains across all stimulus conditions are compared by computing the squared difference between pairs (red lines) and summing across time. Spike trains from the same stimulus conditions (top-left) are typically more similar, leading to smaller distances than pairs from different stimulus conditions (bottom-left). Computed across all spike trains, this yields a matrix of all distances (right). For a given modulation frequency = *ω*, classification performance is measured by selecting a spike train at random and comparing it with a random spike train for all stimulus conditions (omitting the selected spike train). The pair with the minimum distance is then chosen by the classifier. The example distance matrix is for the neuron in Fig 2. Warmer colors indicate larger differences between spike-train pairs.

The responses to tones that were sinusoidally amplitude-modulated (AM) at different modulation frequencies (*f*_*mod*_) were analyzed. Except where stated we present data where the amplitude of the tone is reduced to zero in between the peaks (100% modulation). The temporal aspects of the discharge patterns were originally summarized using the vector strength (VS) metric [24] (see Materials and methods) and fully reported elsewhere [6]. Here, we use an alternative method to quantify the fidelity with which the frequency of the AM stimulus envelope was encoded in spike times. Our approach makes fewer assumptions about the way that information is encoded in spike timing than Vector Strength. Vector Strength assumes that all the information about envelope frequency is encoded by a neuron’s preference to fire around a single phase of the envelope period. For example, if a neuron fires precisely at two different phases during the period of an envelope modulation, VS will be lower than if the neuron only fired at one of those phases [see Laudanski and colleagues (2010a)]. In principle both modes are equally valid ways to encode the signal. A spike train classification algorithm (Fig 1) was used to classify each spike train according to envelope frequency, by measuring its similarity to every other spike train in the dataset [36] (see Materials and methods). The timing of spikes from responses to the same stimulus (Fig 1, top left) tends to be more similar than those from different stimuli (Fig 1, lower left). This classification process compares differences in absolute spike time, was naïve to the stimulus modulation frequency, and was sensitive to any differences in the timing of spikes across individual spike trains.

**Fig 2:**
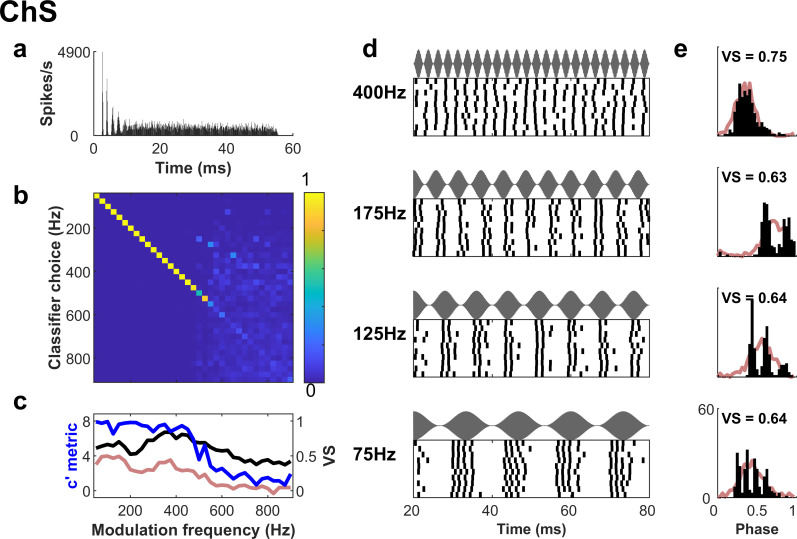
Regular spiking neuron (sustained chopper) that exhibits accurate identification of envelope frequency from its spike trains. **a.** PSTH of the response to a pure tone at the characteristic frequency of the neuron. **b.** Spike train classifier decisions represented as a confusion matrix. Warm colors along the diagonal indicate a large proportion of individual spike trains were assigned to the correct modulation frequency. **c.** Classification performance, expressed as *c′* (*c-prime*; blue), has a low-pass shape across modulation frequency. Vector strength (black) displays a band-pass shape across modulation frequency, characteristic of this neuron type. Red line indicates the *c′* for a simulated “control” neuron which has the same VS as the neuron, but which behaves like a modulated Poisson process (see text). **d.** Raster plots of the responses to amplitude-modulated tones, for several modulation frequencies (stimulus waveforms displayed above each panel). The number of spikes in each stimulus period decreases as the modulation frequency is increased. **e.** Period histograms, folded across stimulus periods, indicate reliable firing at certain phases of the stimulus cycle. Red lines indicate the period histogram for the simulated control neuron.

Fig 2 shows an example of a ChS neuron, a sub-type associated with enhanced envelope coding, with spike timing that is selective for specific modulation frequencies. The ChS neuron displays a regular ‘chopping’ response to a pure tone, as shown in Fig 2a. Fig 2b shows the confusion matrix (with errors represented by non-zero off-diagonal elements) for the output from the spike train classifier applied to this ChS neuron. The confusion matrix is derived from the similarity matrix in Fig 1, which quantifies the differences between all pairs of spike trains. The degree to which each modulation frequency could be identified was summarized using a logistic *softmax* model [37] which yielded a metric, *c′*, for each modulation frequency in a given dataset, yielding a modulation transfer function (MTF-*c′*) summarizing classification performance across modulation frequency. The classification metric, *c′*, quantifies how well modulation frequency can be identified from a set of spike trains where modulation frequency is varying, in a manner which is robust to biases in classifier choice (see Materials and methods and Fig A in S1 Text). The *c′* metric is also less affected by the number of modulation frequencies tested than raw measures of choice probability. The responses of this ChS neuron were sufficient to identify the modulation frequencies below 400 Hz with almost perfect accuracy (the blue line in Fig 2c). A peak *c′* value of 8 is the maximum possible in the analysis (corresponding to ~98% correct for a set of 35 different modulation frequencies). This descends to close to zero above 600 Hz, which corresponds to chance performance.

**Fig 3:**
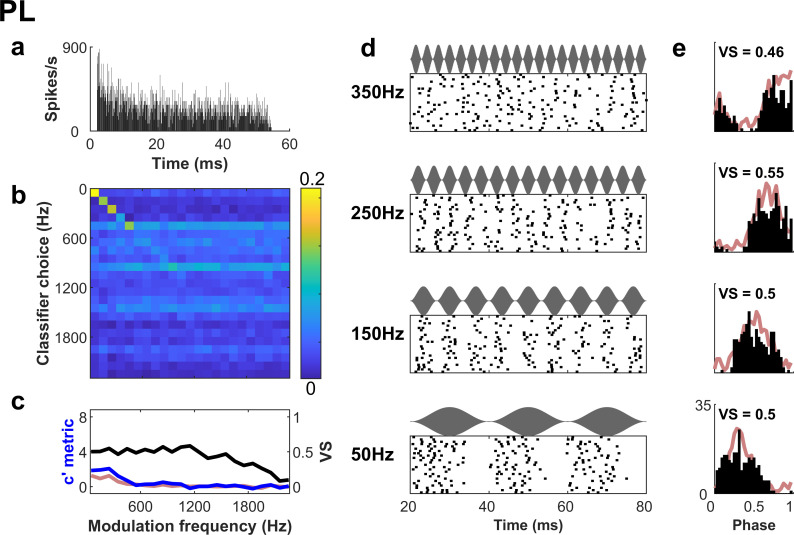
Phasic spiking neuron (primary-like) that displays a preferred phase but little or no additional temporal structure and exhibits poor identification of envelope frequency from its spike trains. **a.** PSTH of response to a characteristic frequency pure tone. **b.** Spike train classifier performance is poor compared to the regular spiking neuron in Fig 2. **c.** Vector strength is a low-pass function of modulation frequency, characteristic of phasic spiking neurons. *c′* is close to chance for most modulation frequencies, indicating poor classification performance. **d.** Raster plots show that the spike timing is markedly less reliable than the sustained chopper neuron example (Fig 2). **e.** Period histograms show a single broad peak at each frequency and the vector strength remains relatively constant. Red lines indicate the behavior of a simulated “control” neuron matched to this primary-like neuron for vector strength but which behaves like a modulated Poisson process (see text).

Fig 3 shows an example of an irregularly-firing PL neuron, a sub-type which is not considered to be specialized for modulation coding. The PL neuron did not demonstrate the regular chopping observed in the pure-tone response of the ChS; its pure-tone response (Fig 3a) resembled that of auditory nerve fibers. This neuron showed some capacity to classify the lowest modulation frequencies (Fig 3b, 3c), and was therefore technically low-pass in its modulation tuning. However, in contrast to the ChS neuron, the PL neuron exhibited poor overall identification of modulation frequency (the peak *c′* of ~2 corresponds to ~25% correct, and drops to close to zero for most modulation frequencies; Fig 3b, 3c), reflecting the trial-to-trial variability of spike timing (Fig 3d). This example neuron also shows a clear tendency to choose certain modulation frequencies irrespective of which one was presented. The *softmax* analysis factors this “bias” out, whereas the raw probability of choosing a given stimulus when it is presented (hit-rate or recall) would have led to the impression that identification was above chance for all modulation frequencies tested (Fig Ab in S1 Text).

The classifier performance was quite different to the Vector Strength metric (VS:24), which has been previously used to quantify the preference of a neuron to fire at a particular phase of a modulation envelope (phase-locking). VS-based modulation transfer functions (MTF-VS) were a poor predictor of classification performance in either of the example neurons (black lines in Figs 2c, 3c show VS). In the PL neuron, spikes occurred at a broad range of phases at a given modulation frequency (VS ~0.5, Fig 3c, 3e) resembling the envelope shape. Yet this neuron performed close to chance (*c′* = 0) at modulation frequency identification. For the ChS neuron, VS was a band-pass function of modulation frequency with a peak near 400 Hz (Fig 2c)—consistent with previous reports. This VS peak corresponds to around 1 spike per stimulus period. However, the modulation frequency with peak VS did not correspond to the frequency of the maximum classifier performance (Fig 2c), which was flat below 400 Hz, and still maximal for the lowest frequency tested (25 Hz). The reason for the difference between measurements for this neuron is that the classifier is sensitive to any reliable difference in the spike trains across modulation frequency. At low modulation frequencies the ChS neuron fired reliably at multiple spikes per envelope period (Fig 2d, 2e): mode-locking. This reliable timing results in very similar spike trains in response to a given stimulus, even though the overall spread of these spikes across the modulation period limits the values of VS. Vector strength has a theoretical maximal value of 1 when a neuron fires at a single preferred phase. If the neuron fires at multiple phases in the modulation cycle, then VS < 1 even if the timing is extremely reliable (in Fig 2, VS ~ 0.64 below 200 Hz).

The classifier reveals that there is useful information about low modulation frequencies in the spike timing of the ChS neuron, to which VS is less sensitive. To quantify the value of this spike-timing information relative to phase-locking, we simulated the neuronal responses that would be expected if VS represented a good description of spiking behavior. This “control” simulation generated spikes from an inhomogeneous Poisson process, which was phase-locked to the envelope and had identical VS values to the data (see Materials and methods). For the control simulation of the example ChS neuron, envelope classification (red line in Fig 2c) was considerably poorer than for the real neuron. The period histograms (PHs) of the simulation (red lines in Fig 2e) reveal a similar general phase of firing but lack the regular structure of the real neuron. In contrast, the classification of the control simulation of the PL example was very similar to the data (red lines in Fig 3c, 3e). It is clear that in *some* neurons, there is information about modulation frequency in the timing of spikes which is not attributable to phase-locking to the envelope frequency.

### Classification-based modulation transfer functions are low-pass but differ in overall performance

**Fig 4:**
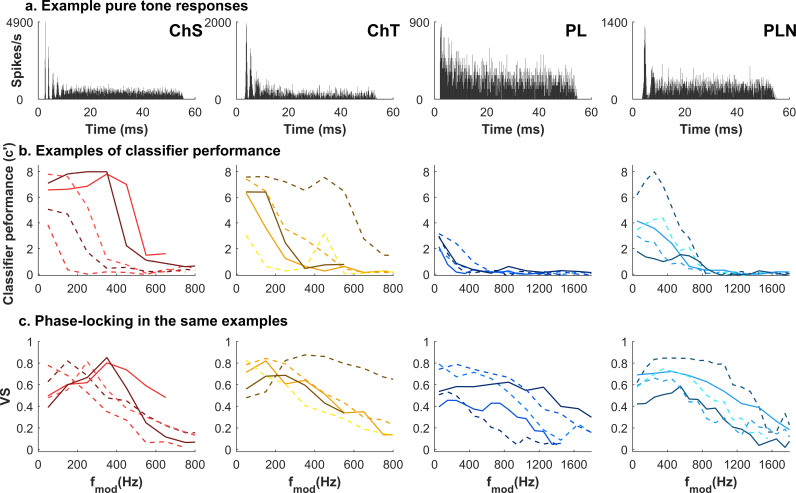
Examples of modulation transfer functions in individual units. Each column shows a different response type and each line style represents an example neuron. **a.** PSTH from a single neuron in response to a characteristic frequency pure tone. **b.** Modulation transfer functions of example neurons of the type shown in **(a)**, expressed in terms of *c′* (MTF-*c′*), and **c.** Phase-locking to the modulation frequency (MTF-VS) from the same data shown in **(b)**. ChS, Sustained chopper; ChT, Transient chopper; PL, Primary-like; PLN, Primary-like notch.

Individual CN neurons of different response types exhibited a range of modulation classification performance. Fig 4 shows example neurons from those types which make up the majority of the data (ChS, ChT, PL, PLN), selected to display a range of *c′* and VS functions (rather than being representative of the population). The classification transfer functions (MTF-*c′*; Fig 4b) were generally low-pass in all four neuron types, and classification was often best at or close to the lowest modulation frequency tested (usually 50 Hz). Yet differences are evident between neuron types, for example, ChS neurons yield much better classification than PL neurons. MTF-VS, in contrast, varied in function shape more than peak VS values (Fig 4c). Notably, these individual ChS neurons phase-locked (VS; Fig 4c left panel) preferentially to different modulation frequencies, as reported previously, yet their classifier functions (*c′*) were low-pass, indicating that classification was best for the lowest modulation frequencies tested. Other quantifications of classifier performance, based on the proportion of correct responses (hit rate/recall accuracy, the harmonic mean of hit rate and correct rejection rate), which did not account as well for factors such as biases in classifier choice and the influence of the number of modulation frequencies tested (see Materials and methods), nevertheless yielded similar results (see Figs I and J in S1 Text).

**Fig 5:**
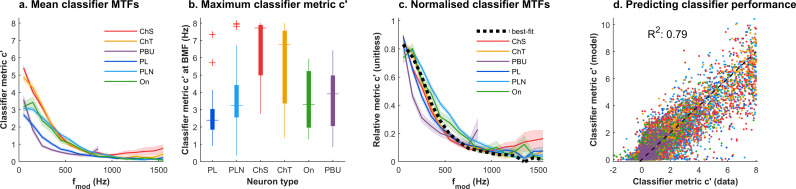
Homogeneity of modulation classification function shapes. **a.** Mean MTF-*c′* for all functions collected for each neuron type. Shading indicates 95% confidence intervals. **b.** Box plots of *c′* at the peak of each modulation transfer function (i.e., BMF-*c′*) for each neuron type, at the lowest sound level tested in each neuron. **c.** The mean MTF-*c′*s, normalized to the maximum mean *c′* for each of the functions in **a**. The dashed black line shows overall mean, which is used for predicting individual *c′*s. **d.** Scatter plot of every *c′* value in the data, compared to the predicted *c′* when modeled as a scaled function of the mean line in **c**. The underlying data for Fig 5b is contained in S1 Data.

Across the population of neurons, the mean population MTF-*c′* was a largely low-pass function of modulation frequency for every neuron type (Fig 5a). There were, nevertheless, systematic differences in *overall* peak classification accuracy (*c′*_*max*_ for each MTF-*c′*) across neuron types (Fig 5b; ChS > ChT> On~PLN > PL ~ PBU), although there was also considerable variation within each type.

To visualize the differences in MTF-*c′* shape, functions from individual neurons were normalized to their peak, maximum *c′* value (Fig 5c). This confirmed that modulation identification in the different neuron types had, on average, a similarly shaped dependence on modulation frequency. This peak normalization analysis included the responses at different overall sound levels (30–70 dB SPL; which are analyzed in more depth below), and additional data where the modulation depth was manipulated (usually 50% or 200%, [31]), and so a similar shape of modulation transfer function was observed across stimulus conditions.

The similarity of the mean modulation transfer function shapes for each neuron type could indicate that the main difference between the MTF-*c′* functions across all neurons individually might be a scaling of a single low-pass function of modulation frequency. Alternatively, averaging out within each neuron type might obscure differences between individual neurons. To distinguish between these possibilities, we sought to quantitatively test how well a single low-pass function could account for the shape of the MTF-*c′* in individual neurons, in all stimulus conditions. First, we calculated a normalized function of the variation of *c′* with the absolute value of modulation frequency, Φ(*f*_*mod*_), which best described the average *shape* of the modulation transfer functions across the entire dataset (black dashed line in Fig 5c; also see Materials and methods). This function is related to each individual MTF-c′, at each level and modulation depth in each neuron, by a best-fitting scaling factor which is applied uniformly to all modulation frequencies in that MTF. Multiplying Φ(*f*_*mod*_) by a scaling factor yields predicted c′ values for an individual MTF (Equation 7). Across the entire dataset, this model predicted individual *c′* values with an *R*^2^ of 0.79 (Fig 5d shows the predicted *c′* values against the data).

**Fig 6:**
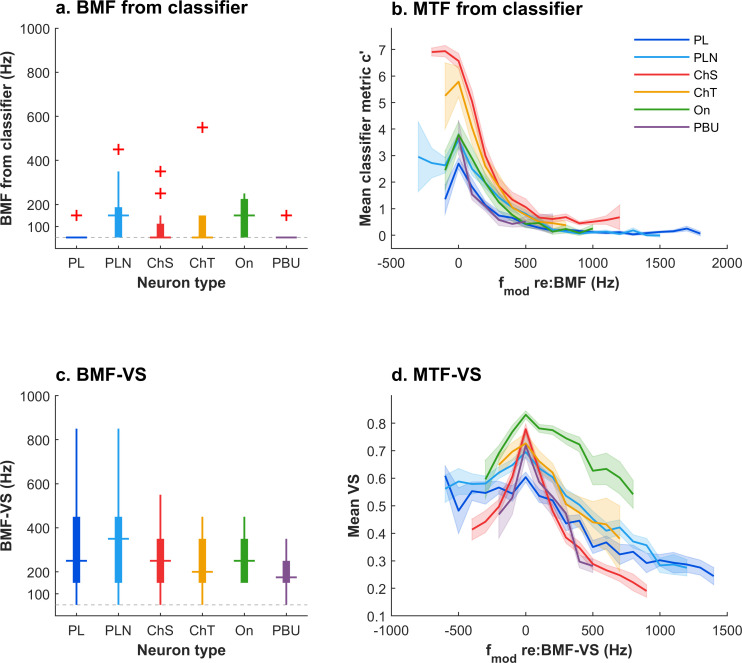
Population Modulation Transfer Functions for each neuron type, derived from a spike timing classifier (MTF-*c′*) or Phase-locking (MTF-VS). **a.** Box plots of the BMF-*c′* for each neuron type. **b.** Mean population modulation transfer function for *c′*, for each neuron type separately, after aligning the BMF for each neuron. One modulation transfer function was selected in each neuron, at the lowest sound level tested. Shaded areas show standard error of the mean. **c, d.** Corresponding population mean modulation transfer function and statistics derived from phase locking (MTF-VS). The underlying data for Fig 6a and 6c is contained in S1 Data.

The (21%) variance unaccounted for by the model was presumably additional variation in the shape of the MTF-*c′*, evidence of which can be seen in the single neuron examples presented (Figs 2–4). To quantify further the variation in modulation transfer function shape, we measured the modulation frequency where classification performance was best in each neuron (Best Modulation Frequency; BMF-*c′*). To minimize the variability due to sound level, we focused here on the lowest sound level available for each neuron (37 dB ± 12 dB, 90% were at 50 dB SPL or lower). The BMF-*c′* reliably occurred at low modulation frequencies (Fig 6a; BMF-*c′* was < 200 Hz in 84% of modulation transfer functions; mean: 148 Hz). BMF-*c′* was often 50 Hz (64%), which was the lowest modulation frequency tested in most neurons. Fig 6b shows the mean MTF-*c′* functions for each type after frequency normalizing each unit to their BMF-*c′*, effectively shifting the functions of individual neurons so that their maxima coincide. Here, small peaks are evident in the functions. However, the frequency normalization process means that the sample of neurons is different at each point on the plot. The low-frequency tails reflect only the neurons where the numerical maximum is other than the lowest frequency tested. These tails were rare in most neuron types (c.f. BMF-*c′* in Fig 6a), with the exception of PLN and onset neurons.

In comparison, our analysis of VS was consistent with previous observations [5,6,14]. Best modulation frequencies based on VS (Fig 6c; BMF-VS) varied over a wider range than those based on classification accuracy (Fig 6a; BMF-*c′*). BMF-VS was concentrated (61% of MTFs) in the range 200–800 Hz (mean BMF-VS: 289 Hz), but this range depended somewhat on neuron type, where the shallow modulation tuning of PL and PLN types was reflected in a larger range of BMF-VS values than other unit types (Fig 6c). Normalized to their BMF-VS, chopper neurons (sustained and transient) showed pronounced peaks and bandpass tuning for MTF-VS, whilst PL neurons were more bandpass in shape. Notably, onset neurons (*n* = 21) had the highest overall VS (consistent with previous studies, 19, 29, 34), yet their classification performance (*c′*) was relatively poor (Figs 5a, 6b), indicating that although onset neurons fire precisely, they do not fire reliably on every modulation cycle (they did not “entrain”). In this respect, rank order of peak VS values and *c′* performance were fairly similar (Fig 6d; On > ChS > ChT > PLN > PL ~ PBU), although classification performance was better for chopper neurons than for onset neurons.

Overall, these results suggest that the main difference in modulation frequency identification between neuron types and individual neurons of each type lies in the overall (and maximum) performance, and not in the relative shapes of the MTF-*c′*, which are predominantly low-pass.

### Modulation frequency classification in individual neurons depends on sound-level

The effects of level on neural modulation classification are of interest because perceptual sensitivity to modulation is either relatively unaffected by sound level (except near to the threshold of audibility), or improves with increasing sound level [22], whilst physiological studies report that VS drops with increasing sound level [4,6,38]. However, as we have seen, VS does not capture all aspects of spike timing. For simplicity, the previous analysis did not describe the influence of stimulus conditions other than modulation frequency. However, data from a range of signal levels and modulation depths were included in the predictive model described above, which implies that the dependence on these conditions can also be characterized as scaling changes. Therefore, we next consider in more detail the effects of signal level.

**Fig 7:**
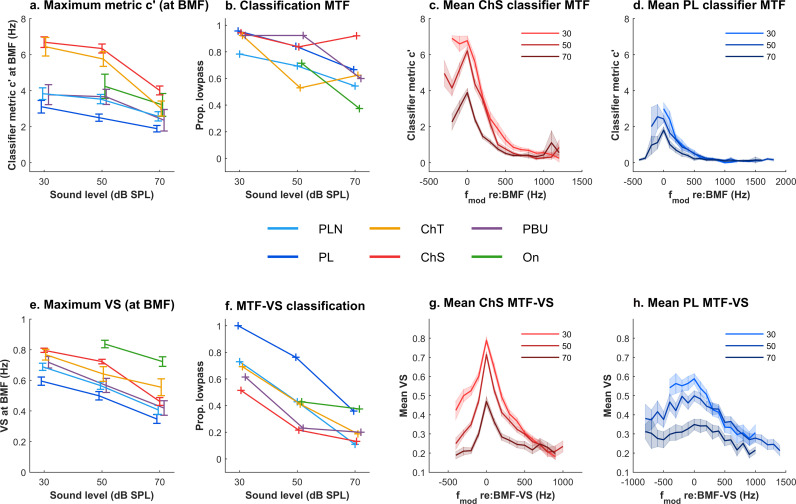
Modulation Transfer Functions as a function of sound level, derived from a spike timing classifier (MTF-*c′*) or Phase-locking (MTF-VS). **a.** Maximum *c′* at the peak of the corresponding modulation transfer function, split by neuron type, and sound level. **b.** Proportion of MTF-*c′* shapes classified as lowpass (criteria described in methods), split by neuron type and sound level. **c.** Mean population MTF-*c′* for sustained chopper (ChS) neurons as a function of sound level. **d.** Mean population MTF-*c′* for primary-like (PL) neurons as a function of sound level. **e.** Peak vector strength values and **f**. proportion of lowpass MTF-VS as a function of neuron type and level. **g.** Mean population MTF-VS for sustained chopper neurons as a function of sound level. **h.** Mean population MTF-VS for primary-like neurons as a function of sound level.

Peak *c′* decreased systematically with sound level (Fig 7a shows all neuron types for a modulation depth of 100%; Kruskal-Wallis non-parametric ANOVAs for peak MTF-*c′*, χ^2^ (2,488) = 77.3 *p* < .0001), as previously reported for VS (and shown in Fig 7e). For both *c′* and VS, there was also a decrease in the proportion of lowpass functions with increasing level (Fig 7b, 7f; χ^2^ (2,488) = 27.6, *p* < 0.0001 for *c′* and χ^2^(2,505) = 80.88, *p* < 0.0001 for VS). However, there were more low-pass MTF-*c′* functions than low-pass MTF-VS functions across all neuron types (χ^2^(1, *N* = 117,150,134) = 11.8, 31.7, and 65.6 at 30, 50, and 70 dB SPL with *p* < 0.0001 at all signal levels) and for each neuron type individually (χ^2^ (1,120) = 14.0, *p* = 0.0002 for PLN; χ^2^ (1,106) = 89.7, *p* < 0.0001 for ChS; χ^2^ (1,39) = 5.4, *p* = 0.0202 for ChT; χ^2^ (1,34) = 20.1, *p* < 0.0001 for PBU), except for onset (χ^2^ (1,13) = 0.04, *p* = 0.84) and PL neurons (χ^2^ (1,87) = 3.0, *p* = 0.08), consistent with previous observations that MTF-VS is lowpass in these neurons [5,6]. The proportions of different shaped MTF-VS functions depended on the neuron type, and there was a strong shift towards bandpass functions with increasing signal level. The differences between *c′* and VS were also evident in mean population modulation transfer functions at different sound levels, which are again shown relative to best modulation frequency for comparison between *c′* and VS: consistent with the predictive model, MTF-*c′*s for ChS and PL neurons were predominantly low-pass (Fig 7c and 7d; the low-frequency tails are attributable to a minority of neurons, see Fig 6), whereas mean MTF-VS was robustly bandpass in shape for ChS (Fig 7g), but low-pass for PL neurons (Fig 7h).

These findings also held for other modulation depths, and when there are smaller differences between modulation frequency. At 200% modulation (shown in Fig B in S1 Text), there are again more lowpass MTF-*c′* functions than MTF-VS functions. 200% modulation stimuli are characterized spectrally by having three frequency components of equal amplitude, whereas 100% modulation stimuli are characterized by a stronger peak at the carrier frequency. The transfer functions of 200% modulations derived from VS (MTF-VS) were bandpass even in PL neurons which have predominantly lowpass MTF-VS functions at 100% modulation (Rhode 1994). Yet, modulation classification functions (MTF-*c′*s) were robustly lowpass across all neuron types, and both classification performance and VS were reduced with increasing signal level. There were fewer data at modulation depths of less than 100%. Classifier performance generally dropped as modulation depth decreased (Fig C in S1 Text), but MTF-*c′* functions were nevertheless predominantly low-pass. Modulation classification in ChS neurons was also low-pass, and generally reduced with sound level, when the difference between modulation frequencies was smaller (25 Hz; Fig Da in S1 Text), though our sample of neurons was small (7 ChSs). Modulation classification was even stronger and reduced with sound level, in a single ChT neuron which was tested for 5 Hz steps at modulation frequencies below 50 Hz (Fig Db in S1 Text). Thus, in so far as we are able to determine, these results were robust across a wide range of envelope characteristics.

Based on the consistent effects of sound level and modulation depth, we asked what proportion of the observed modulation classification might be explained by stimulus conditions alone. To do this we used a regression model where, instead of fitting each modulation transfer function individually, stimulus conditions alone determined the scaling factor for Φ(f_*mod*_) (Equation 8; see Materials and methods). Thus, the model predicts identical performance for all modulation transfer functions with the same sound level and modulation depth. A model with four free parameters was able to predict 45% of the variance of individual *c′* values across the entire dataset. We also tried adding further free parameters (5; see Equation 9 in Materials and methods) to enable the model to account for differences between neuron types. Only a further 2% of the variance (i.e., *R*^2^ = 0.47) was accounted for, implying that a sizable amount of the variance in identifying modulation frequency is common to all CN neurons (perhaps inherited from the cochlea) and depends on stimulus conditions more than on neuron type.

### Modulation frequency classification at high sound levels is robust in small populations of high-performing neurons

Whilst the low-pass characteristics of modulation classification functions more closely align neural coding with perception, the systematic decreases of neural modulation classification with increasing sound level appear at odds with perception [23]. We investigated the identification of modulation frequency in small populations of neurons by extending our existing classifier, so that it functioned with multiple neurons. The method is one commonly used to study population coding with spike-distance metrics, which maintains the identity (i.e., “a labeled line code”) and the timing information of each neuron [39].

**Fig 8:**
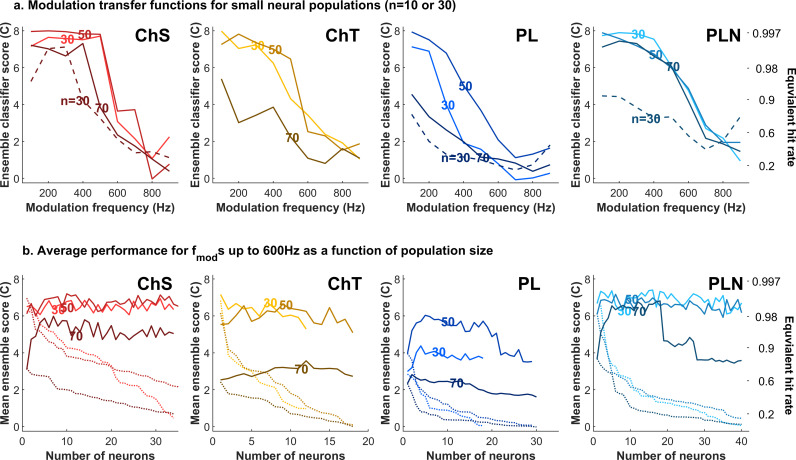
Modulation classification and the variation in sound level, from small populations of neurons. **A.** Modulation transfer functions for ensembles of each neuron type at different sound levels. Solid lines show classifier performance for 10 neurons with the best individual performance for each neuron type and sound level (unit is dB SPL). Dashed lines show the performance at 70 dB for the 30 best-performing neurons (not available for transient choppers). N.B. Onset and pause-buildup neuron populations not shown due to the small number of neurons. In the right-hand panel, the equivalent hit rate is also shown, for comparison. This scaling holds for all panels. **B.** Mean classifier performance (modulation frequency <600 Hz) as a function of the population size used for classification, in order of decreasing performance for the individual neurons (so best performing first). Solid lines show population classifier performance, whilst the dotted lines show the mean performance of the worst neuron in each set of neurons.

Fig 8a shows MTF-*c′*s of small (*n* = 10 or 30) populations of neurons of a given type, selected as the highest performing (mean *c′*) neurons in the available sample. Populations of ChS neurons supported modulation classification that was markedly more stable across sound level than individual neurons. Small populations of PLN neurons were even more robust to level changes, with *c′*s for clusters of 5–15 neurons differing by a value of 1 or less across the 40 dB range tested. In contrast to ChS and PLN populations, classifier performance in both ChT and PL populations dropped markedly with increasing sound level. As in individual neurons, population modulation transfer functions were predominantly low-pass in shape. The level-robustness is also observed if instead we consider classifier hit-rate to quantify performance, or F1-score (see Figs I and J in S1 Text).

In order to determine whether performance reflected coding in high-performing individual neurons or an even distribution of information across the populations, we systematically varied the sizes of the populations used for classification. Fig 8b shows the mean performance (for *f*_*mod*_ ≤ 600 Hz) as a function of population size, adding neurons in order of decreasing individual performance. This mean performance revealed further differences across neuron type and sound level. Sustained chopper population performance at low and medium (50 dB SPL) levels was only slightly higher than that of the best-performing neurons, while at 70 dB SPL, performance effectively doubled as the population size increased from 1 to 10 neurons. In other neuron types, performance increased more gradually with increasing populations at all sound levels, indicating that information was distributed across a number of neurons. In PLN neurons, population choice was critical: a small population (~5 to 15) of the highest performing neurons provided for reliable identification at 70 dB SPL, but performance dropped in larger populations. This is in contrast to populations of ChS neurons, where the inclusion of neurons with poor individual performance (dashed line shows performance of the last added and worst neuron) did not harm the ensemble performance.

Thus, small ensembles of neurons in the ventral CN can support the identification of modulation frequency at a wide range of sound levels. The level robustness, and the dependence on the selection of the sub-population depends somewhat on neuron type, with a small minority of PLN neurons providing the best performance. Sustained chopper neurons are almost as good as PLN neurons, but adding poorer performing neurons to the cluster does not harm performance. Interestingly, this implies that level-robust performance can be achieved downstream by integrating inputs from ChSs indiscriminately, whereas it would require selecting inputs only high-performing PLN neurons.

Overall, spike timing in the auditory brainstem supports the coding of modulation frequency, with a low-pass transfer characteristic. Whilst classification is accurate in individual neurons at low sound levels, classification at high sound levels remains robust in small populations of high-performing ChS and PLN neurons.

### Reliable spiking and mode-locking are hallmarks of good envelope coding

**Fig 9:**
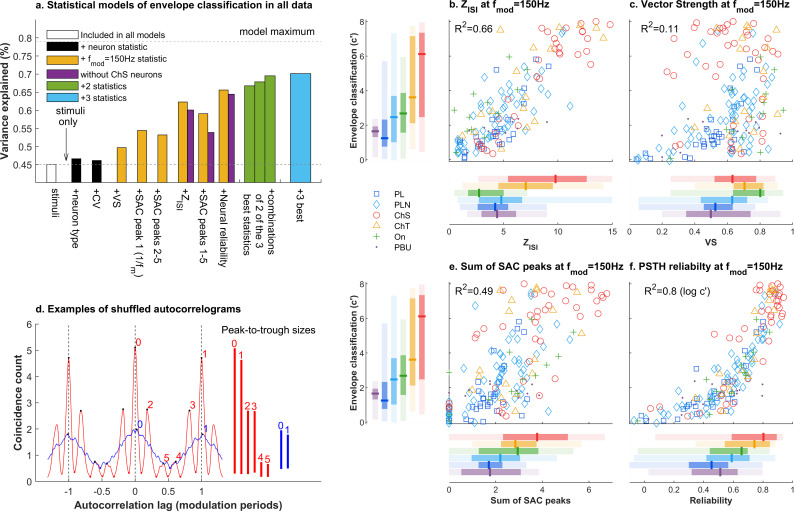
a. How various statistical models account for modulation classification performance across the entire dataset. Each model has stimulus conditions plus one or more additional parameters as predictors of *c′*. Parameters are *Type:* neuron classification (primary-like, sustained chopper, etc.); *CV:* Coefficient of variation of the interspike intervals in response to a pure tone at CF. *VS:* Vector Strength; *SAC peak 1:* The height of the peak in the shuffled autocorrelogram (SAC) at the modulation period (1/modulation frequency); *SAC peaks 2-5:* The heights of the next 4 largest peaks in the SAC, between the zero-lag peak and the modulation period; *Z*_*ISI*_: A Z-scored measure of the degree to which interval statistics differ from that predicted from the phase statistics alone; *SAC peaks 1-5:* The five largest peaks in the SAC, where peak 1 is the modulation period; *Neural reliability*: A measure of the reliability of the PSTH in response to the amplitude-modulated tone. A single predictor (the statistic specified in the panel title for *f*_mod_ ~150 Hz) is used at all modulation frequencies for each dataset. Purple bars show fits to the data with sustained chopper neurons excluded. **b.** How the *Z*_*ISI*_ statistic is related to *c′* for a modulation frequency of ~150 Hz; Horizontal sidebars show the median, 50% and 95% ranges for *Z*_*ISI*_ and vertical side bars show this for *c′*; **c.** As **b** for Vector Strength; **d.** Examples of the shuffled autocorrelation functions for the sustained chopper neuron in Fig 2 (modulation frequency = 125 Hz) and primary-like neuron in Fig 3 (modulation frequency = 150 Hz). Numbered peaks indicate the significant peaks up to the lag which equals the modulation period, according to the peak picking algorithm (at *p* < .0001). Vertical lines show the peak-to-trough heights extracted for each significant peak, in order of decreasing value; **e.** As **b** for the sum of peaks 1–5 in the shuffled autocorrelation. **f.** As **b** for the reliability of the PSTH. In **f** *R*^*2*^ is calculated by taking the log of *c′*. The underlying data for Fig 9a is contained in S2 Data and the underlying data for Fig 9b, 9c, 9e, and 9f is contained in S3 Data.

The best classifier performance was observed in ChS neurons, the most regular spiking type of CN neurons. But neuron type was generally a poor predictor of classification performance, and there was great variation in performance within neurons of a given type. So, what aspects of spiking are important for supporting good identification of envelope shape? To investigate this, we extended the non-linear statistical regressions of *c′* by adding various quantifications of spiking behavior as additional predictors, with each quantifying a conceptually distinct aspect of spiking behavior (Fig 9a).

One hypothesis is that mode-locking spiking patterns, seen in our example ChS neuron (Fig 2), might contribute positively to neural coding. Mode-locking is a term originating from non-linear oscillator theory, reflecting varied “modes” of oscillation displayed when driven by a periodic input [26]. Different modes arise from an interaction between the dynamics of the input, here the varying sound envelope, and the intrinsic dynamics of the oscillator, which here would be neuron properties such as the time constant of integration associated with membrane capacitance, ionic currents, and the abrupt hyperpolarization following an action potential [40]. The outcome of this interaction is that spike times depend strongly on both the envelope and the time of the previous spike. This spiking behavior stands in contrast to a simpler model of spiking behavior, where consecutive spikes occur independently as a simple probabilistic function of the envelope, i.e., a Poisson process.

Motivated by the theoretical models, we sought to quantify the spiking dynamics which are likely to give rise to mode-locking. A hallmark of a neuron behaving as a modulated Poisson process is that the spike-interval statistics follow directly from the modulations in firing rate: randomly redistributing spike times across modulation periods whilst maintaining their phase (position within the period) should not affect the interval structure. However, if spike timing depends both on envelope phase and the time interval since the previous spike, then such a shuffling of the spike times will affect the interval structure. A metric, Z_ISI_ [28], was used to quantify the changes in interspike intervals (ISIs) caused by this ‘phase-shuffling’, with increasing values indicating a greater dependence of each spike on the timing of the previous spike (see Materials and methods).

Applied to the current dataset the Z_ISI_ revealed a hierarchy across neuron types (Fig 9b; horizontal bars; ChS > ChT > PLN > PBU ~ PL>On; also see Fig E in S1 Text). Regular firing neuron types (e.g., ChS, ChT), which displayed good modulation classification, also scored highest on Z_ISI._ Also, Z_ISI_ and *c′* were strongly correlated at low modulation frequencies in individual neurons (shown for a modulation frequency of 150 Hz in Fig 9b). To quantify this relationship further, we extended the non-linear statistical regressions of *c′* by adding Z_ISI_ as a predictor to the previous regression model, which otherwise included only sound level and modulation depth as predictors. The addition of the Z_ISI_ statistic markedly improved the predictive power across all modulation frequencies (62% versus 45% for stimuli alone; Fig 9a). This improvement largely held even when the most regular neuron type, ChS neurons, were removed from the data (60% of variance). The regression model employed a single Z_ISI_ measure at a modulation frequency of *~*150 Hz as the predictor for each dataset and applied to all modulation frequences. This choice has several advantages and does not influence the overall conclusions (see S1 Text for an in-depth analysis of this choice). The analysis supports the hypothesis that the dynamic behavior of neurons, which underlies mode-locking in theoretical models, contributes to good envelope coding in CN neurons of all types. In contrast to Z_ISI_, VS was poorly correlated with classifier performance, whether at individual frequencies (Fig 9c) or as a predictor in a regression model (50% versus 45% for stimulus conditions alone; Fig 9a).

To unpack further the role of mode-locking, we sought to determine whether the mode of firing itself contributed to classifier performance. The Z_ISI_ statistic quantified a property of neurons which is likely to lead to mode-locking, but it did not explicitly test whether a neuron fires in modes other than those which are approximated by phase-locking (1-*q*; 1 spike every *q* periods with *q* being any integer). However, identifying modes precisely in real neurons is challenging due their stochastic behavior (unlike in models where noise can easily be removed). To quantify spiking modes in the face of this stochasticity, we computed the shuffled autocorrelogram (SAC) of each response, which counts ISIs between spike trains using autocorrelation (see Materials and methods). Because this analysis operates across trials, and between non-adjacent spikes as well as adjacent spikes, it is statistically powerful and is therefore very sensitive to temporal structure. Fig 9d shows SACs from the example neurons in Figs 2 and 3. Modes of spiking other than 1-*q* (which is how phase-locking appears in the SAC) show up as peaks in the SAC at delays other than integer multiples of the envelope period, and are clear from the response of the ChS neuron (red), but absent from the response of the PL neuron (blue). We found that additional peaks were common in the SAC at low modulation frequencies in ChS neurons (~45% of the population for modulation frequencies < 400 Hz) and PBU neurons (~30%), but rare in other neuron types (Fig G in S1 Text). We quantified the peaks in the SAC using a peak-picking algorithm, which yielded the amplitudes of significant peaks (at *p* < .0001) relative to the nearest local minima (Fig 9d shows peak-to-trough sizes as vertical bars; see Materials and methods). A simple unweighted sum of the significant peaks correlated well with envelope classification of low modulation frequencies (Fig 9e).

We quantified the predictive power of different peaks in the SAC across the entire dataset by adding the amplitude of each peak at a modulation frequency ~150 Hz as parameters to the stimulus-only regression model. Adding in only the peak at the modulation period, which is expected from phase-locking to the envelope, resulted in an increase in predictive power (54% of variance versus 45% for stimulus alone). Adding in the additional peaks (2–5; and a value of zero where there were no peaks) as well as the peak at the modulation period resulted in a further increase in variance explained (59%). The difference in variance accounted for between these two models (5%) quantifies the value of reliable inter-spike intervals at less than the modulation period. Thus, the difference in variance offers an explicit test of the value of mode-locking (modes of *p-q* where *p ≠ 1*) over phase-locking (*1-q*).

Adding in only the additional peaks (2–5) and omitting the period peak resulted in a similar predictive power to the period peak alone. Furthermore, omitting the ChSs (in which these additional peaks were observed) entirely nullified the predictive power of the additional SAC peaks (Fig 9a, purple bar for SAC peaks 1–5), consistent with the observation that these peaks primarily occurred in ChS neurons. Yet, this additional temporal structure was not necessary for good identification of modulation frequency: some ChT neurons (Fig 9b, 9c, 9e, 9f; yellow pyramids) and a few PLN neurons (blue diamonds) performed almost as well as the best ChS neurons despite a lack of additional SAC peaks. Thus, additional temporal structure observed during mode-locking contributes positively to envelope coding in ChS neurons, but other coding strategies in other neuron types can result in similar performance.

To test the idea that reliability of spiking, regardless of the specific patterns of spikes, is important for coding of envelope we used a direct measure of the reliability of spike times. This calculation is simply the Pearson correlation of the peri-stimulus time histogram (PSTH) of the responses to odd and even stimulus presentations at modulation frequency ~150 Hz [41]. Consistent with the fundamental importance of reliable spiking, this measure correlates well with *c′* at low envelope frequencies (Fig 9f) and, over the entire dataset, the reliability statistic slightly greater predictive power than Z_ISI_ (66% versus 62%). Another conventional characterization of regularity in CN neurons, the coefficient of variation (CV) of the inter-spike intervals in response to a pure tone, performed poorly (46%) in comparison. A more in-depth exploration of predictors of envelope classification performance is found in S1 Text.

Our results suggest that three properties of spike trains are all associated with good envelope coding. Indeed, we found that SAC peaks 1–5, Z_ISI_ and PSTH reliability were all strongly correlated with each other (Fig K in S1 Text). Adding any two (Fig 9a, green bars), or all three of these statistics (Fig 9a, blue bar) into a single regression model improved the predictions of the models further, but did not indicate superiority of any one statistic. The relative predictive power of all these different models was very similar for other quantifications of classifier performance (see Figs I and J in S1 Text). In summary, reliability and non-Poisson behavior were closely linked characteristics which were important for representing envelope in all neurons. In the most regular spiking neuron type in the CN (ChS neurons) mode-locked patterns of spike timing, distinct from phase-locked behavior, contributed positively to envelope coding. In contrast, statistics which quantified synchronization to the envelope frequency were poor predictors of classification in all neuron types.

## Discussion

### The relationship between neural coding and perception

Spike-timing-based analysis revealed that the best identification of envelope frequency occurred at low modulation frequencies and that regularly-firing chopper neurons showed the best envelope coding overall. Superior classification of low modulation frequencies is consistent with perceptual modulation detection [22] and identification of AM frequencies [42], both of which are excellent below around 300 Hz, and also the ecological predominance of low modulation frequencies [3,17]. In contrast, synchrony-based measures (MTF-VS) in chopper neurons were bandpass tuned and selective for 200–500 Hz modulations, with lower modulation frequencies (<200 Hz) less well represented.

A further property of modulation perception is its robustness to variation in sound level [22]. Our results suggest that single CN neurons are not robust to variations in sound level, either using VS or a spike-timing-based classifier. However, selective pooling of information across neurons was sufficient for accurate identification of envelope up to at least 70 dB SPL. Additional factors are only likely to improve the level-robustness of modulation coding further. Efferent connections to the cochlea and CN, which will be more active in the absence of anesthesia, will act to increase dynamic range in CN neurons [43,44]. Recent evidence has revealed that CN neuron activity is markedly different under isoflurane and ketamine anesthesia (though not pentobarbital as used here), as compared to an awake animal, but that effects at the population level are subtle [45]. In humans, listening strategies may well further augment perception: at high sound levels the recruitment of neurons tuned to characteristic frequencies other than the carrier tone would provide additional modulation information. Recruitment cannot explain the level robustness of modulation perception generally, which remains robust with level for wideband sounds where recruitment of off-CF neurons does not occur, but it is known to contribute to modulation detection in narrowband sounds [20]. Taken together with our observations, there is no reason to think a qualitative discrepancy with perception or sound statistics remains.

Some work remains to reconcile physiology and perception quantitatively, for which differences in experimental methodology and species become more critical. Perception at shallow modulation depths, of which our data contained a small sample, is an example of this. Sayles and colleagues [14] found neural thresholds for the detection of shallow modulations were commensurate with perceptual detection thresholds. Their method was based on VS and therefore yielded bandpass functions in these most sensitive neuron types. Using our method, we would predict similar sensitivity but with a low-pass function. Similarly, although our results hold for small changes in envelope frequency in the data that were available to us, it would be desirable to confirm this in a large population where the minimal discriminable difference in frequency can be determined. A further challenge for quantitative comparisons could result from species differences in cochlear tuning and phase locking, which can influence the shape of modulation tuning functions [46,47]. These nuances could prove difficult to resolve experimentally, but we are optimistic that a carefully constructed simulation could predict human envelope perception with reasonable quantitative accuracy. Such a simulation would use biophysically constrained spiking models of CN neurons [48] driven by cochlear models which can account for species differences in peripheral processing [49] and efferent modulation [50,51], and analyzed using the methods described here.

### Spike-timing codes: Mode-locking and reliability versus faithful synchrony to the envelope

We find that it is not faithful synchrony to modulation frequency which determines how well an envelope is encoded. It is important that neurons fire reliably across trials in response to a given stimulus, but not how envelope information is encoded. “Spike distance metric” methods like those employed here [52–54] can find this information because they make no assumptions about the relationship between the stimulus and the pattern of spikes; only that the timing of spikes depends reliably on the stimulus. In contrast, VS measures how tightly spikes are timed in a unimodal distribution about a particular phase of the modulation frequency.

The CN appears to employ (at least) two different strategies for representing envelope shape. Across all neuron types, envelopes are more discriminable when spike timing is not just dependent on the phase within a modulation period. Indeed, the less spiking behavior conforms to the expectations of a time-varying firing rate, and the more spike timing depends on when the previous spike occurred, the better modulation frequency can be identified. This suggests that the dynamics of the spiking process are having a positive influence on the encoding of envelope.

In ChS neurons, which are the most regular spiking of CN neurons, we find “mode-locking” to low-frequency modulations [28]. Mode-locked spiking patterns have complex phase distributions (see Figs 1and 2), which can yield low VS values. However, these patterns are reliably timed, and we show here that modes of locking other than a 1-*q* relationship with the modulation frequency are correlated with modulation classification performance in these neurons. This provides clear evidence that the degree of synchrony to the envelope frequency does not always accurately estimate the information carried.

A reasonable interpretation of these results might be that the intrinsic non-linear dynamics of neuronal processing [26] in the CN improve the reliability of spiking generally. In neurons where the influence of these dynamics is strong, this can create spike timing patterns which are a complex function of the stimulus envelope, but which nevertheless encodes the envelope accurately. However, we also found that some ChT neurons and PLN neurons support similar levels of classification despite firing less regularly and showing little evidence of mode-locking at ratios other than 1-*q*. Thus, mode-locking is not apparently necessary for good coding of envelope, suggesting that this simple interpretation is incomplete.

The known differences in local circuitry and intrinsic cell properties may go some way to accounting for the multiple effective coding strategies. Sustained chopper and ChT responses are associated with stellate cell (T-stellate) morphology, whilst PLN responses are associated with globular bushy cells [55]. Both cell types receive convergent inputs from auditory nerve fibers [13]. Sustained and ChTs share similar ionic currents and synaptic dynamics [56]. However, ChTs receive fast, timed inhibitory input from D-stellate cells [57]. We speculate that this inhibition may influence the dynamics of the envelope responses in transient-choppers, perhaps preventing mode-locked spike-timing at ratios other than 1-*q*. Globular bushy cell neurons possess a low-threshold Potassium current which produces a strong hyperpolarization following each spike, and a much shorter integration time constant than stellate cells [56]. This short integration time should restrict the influence of one spike on the next to shorter intervals, presumably leading to spiking dynamics which are quite different to ChS (T-stellate) neurons.

### Robust encoding of binaural envelope cues in small ensembles of primary-like-notch neurons

Our results may also clarify the processing of interaural timing delays (ITDs); cues for sound localization in the envelopes of high-frequency sounds [58]. This interaural comparison is believed to be performed by neurons in the lateral superior olivary nucleus (LSO) [59], which receive ipsilateral excitatory inputs directly from PL neurons, and contralateral inhibition via the medial nucleus of the trapezoid body (MNTB) [60]. Primarylike-notch neurons synapse on to MNTB neurons with large single ‘Calyx of Held’ synapses, which provide precisely timed inhibition to LSO neurons [61,62]. Psychophysical and physiological data both demonstrate good sensitivity to envelope ITDs (~100 µs) [63,64] at high modulation frequencies (up to 800 Hz) [65,66], above the limit of CN chopper neurons, or monaural responses of LSO neurons.

Some low-frequency PLN neurons show exceptional phase locking to the carrier [67], presumably to encode low-carrier-frequency ITDs. Our analyses reveal a concomitant result for envelope processing at high-carrier-frequencies in PLN neurons, and that selective ensembles of neurons support envelope coding just as well as ensembles of chopper neurons. This is consistent with the properties of LSO neurons and envelope ITD perception. Simulations also suggest integration of multiple inputs are required for envelope processing in LSO neurons [68]. Thus, a convergence of MNTB neurons, which are driven by these select PLN neurons in CN, could provide precise inhibition to LSO neurons, and support the envelope ITD sensitivity seen perceptually and in the superior olive.

### Modulation processing in the auditory pathway

Our results imply that channels processing different modulation frequencies do not first emerge in CN chopper neurons, as has been proposed [11,69,70]. However, far from bringing our understanding of other data into question, a distributed low-pass temporal code in CN arguably fits better with downstream processing. Envelope encoding is progressively transformed from a temporal to rate code as the auditory pathway is ascended [15,71–74]. In the IC, the firing rates of neurons are often bandpass tuned, but to a range of lower frequencies than the VS-BMFs of CN choppers [11]. Inferior colliculus neurons can also display multimodal, high-pass, and band-reject modulation tuning functions [18,75,76], for which the previously proposed bandpass VS-MTFs of CN choppers were not in any case a logical pre-cursor.

It is also likely that perception does not rely solely on distinct firing rate-tuned modulation channels: rate tuning typically is not robust enough to other stimulus parameters such as envelope shape and sound level [73,77,78]. Temporal coding of low-frequency envelope persists, even at the level of primary auditory cortex [79] and high-rate temporal coding of modulation (~500 Hz) is found at the input to cortical neurons [80]. Clearly, temporal coding of envelope is precise to a few milliseconds several synapses beyond the projections from the CN. In the IC, temporal coding accounts for the perception of envelope better than rate coding, at least in birds and humans [18,81,82], though rabbit’s perception appears to be better explain by rate coding [83]. In addition, in some cortical neurons, firing rates are proportional to modulation frequency [73], rather than forming tuned channels. Thus, the balance of evidence points to a diverse mix of codes across the auditory pathway [11,73,74,84], as in other modalities [85], for which the low-pass distributed temporal code we report in CN is a suitable pre-cursor.

It remains an open question how the information from CN is transformed in the IC. Several computational models have been proposed for transforming synchrony-based spike timing coding of envelope in the CN into rate-place representations of envelope in the IC. Diverse tuning functions could arise in the IC from delayed inhibition [41,86–88]. Alternatively, if chopper neurons (T-stellate cells) with appropriate synchrony tuning converge onto IC neurons, a rate-tuning to modulation frequency can emerge [89]. Modulation tuning can even emerge in artificial neural networks where there is no initial frequency analysis [90]. Unfortunately, although these models demonstrate that the problem is computationally tractable, there is no strong evidence to support any of them in particular.

No computational models have addressed how mode-locked spike patterns could give rise to the modulation tuning seen in the IC. Two of the aforementioned models [87,89] employed integrate-and-fire models of neurons which are likely to mode-lock and it is possible that mode-locking contributed to their function, but this was not considered in those studies. There is, however, good evidence that other mechanisms do exist which could process the envelope information in mode-locked spike patterns. Spike timing-dependent plasticity can rapidly detect arbitrary repeating patterns of spikes, even finding repeated patterns in the presence of additional unrelated spikes [91]. This demonstrates that: (i) Simple neural mechanisms which are known to exist can be configured to be sensitive to spike timing patterns like those seen during mode-locking; (ii) Sensitivity to mode-locked spike patterns can be acquired via an unsupervised learning process.

A question exists as to whether a loss of temporal precision at the next synapse could mean that the detailed timing information of spike trains, whether mode-locked or phase-locked, will be lost. This is not possible to answer given the lack of knowledge about the dynamics of T-stellate synapses. To investigate the theoretical impact of reduced temporal resolution on the spike-time coding in our neurons, we interrogated our classifier results in greater depth. The classifier was run at a range of temporal resolutions, imposed by the smoothing function used to compute differences between spike trains (Fig 1). In our analyses thus far we had chosen the best classifier for each dataset, as a simple way to optimize the classifier across the diversity of neural responses. The temporal resolution which led to the best overall classification performance was most often at one of the two highest resolutions we tested (*τ* = 1–2 ms; Fig L in S1 Text). Instead, fixing the classifier at a much lower resolution (*τ* = 10 ms), we found that classifier performance dropped more rapidly with modulation frequency, but individual functions were still low-pass (Fig M in S1 Text), still depended on sound level in much the same way (Fig N in S1 Text) and the hierarchy of performance across neuron type was maintained. Thus, although a loss of temporal resolution at the next synapse will reduce the information about high-frequency envelopes, it may not impact much on the coding of low-frequency envelopes.

### Wider implications for the processing of complex sounds

Our results lead to a revised view of the role of the brainstem in processing envelopes. This view has wide ramifications for the processing of speech and other environmental sounds, and should enable a more coherent understanding of how information about complex sounds is transformed at the critical point where it enters the central nervous system. A distributed code which represents slow modulations well and is reasonably robust across sound level appears to be fit for the purpose of speech recognition. This remains to be tested, as there are remarkably few studies of the CN responses of modulation-rich stimuli such as natural speech or other vocalizations [16,92].

As the only target of cochlear nerve fibers, the CN is directly impacted by the changes wrought by cochlear hearing loss. One kind of hearing loss, synaptopathy [93], reduces the convergence of auditory nerve fibers onto CN neurons, impairing responses to tones-in-noise [94]. It should also reduce the reliability of envelope coding in chopper neurons [95], leading to less mode-locked spike trains and fewer sustained-chopper responses. Fewer sustained-chopper responses would logically degrade envelope population-coding at high sound levels (c.f. Fig 8) —an effect which is usually attributed to the loss of high-threshold, low spontaneous rate nerve fibers [96].

Elsewhere in the auditory system, mode-locking has been hypothesized to contribute to the processing of pitch and harmony [97]. Our observations might also have implications for processing of low-frequency temporal fine structure, critical for the perception of pitch [98]. Recent data suggest that spherical bushy cells which predominate in the low-frequency rostral pole of the CN [99,100], can show complex modes of firing for temporal fine structure [30]. It is possible that low-frequency neurons (which are problematic to classify) encode temporal fine structure in patterns of spikes [101,102] that phase-locking analysis has obscured.

It is even possible that mode-locking may have an impact on non-invasive measurements of envelope. The frequency-following-response is an evoked potential that can be measured from the scalp, which shows synchronization to the envelopes of sound up to several hundred Hertz. A component of this signal originates from the CN [103], and therefore could be impacted by mode-locking. For example, mode-locking could lead to a reduction in synchrony to the envelope, as it does in ChSs, yielding an underestimation of the envelope information available at a neuronal level.

In summary, our results reveal a code for sound envelope which is more consistent with what is required for perception and the statistics of natural sound than previous accounts, demonstrates the importance of mode-locked spike patterns, and is likely to have wider implications.

## Materials and methods

### Experimental preparation and acoustic stimulation

The animal preparation has been described in detail previously [6,31] along with the original analysis of these data. The protocol for these experiments was reviewed and approved by the Animal Care committee of the University of Wisconsin, which is a PHS Assured, USDA registered, and AAALAC International Accredited Institution (PHS Assurance Number D16-00239 (A3368-01), USDA Research Registration Number A3368). Data were collected from 42 adult cats, weighing between 2 and 5 kg [6,31]. Briefly, animals were anaesthetized with pentobarbital sodium (50 mg/kg) and were maintained in an areflexive state during the course of the experiment. The animal was artificially respired and its body temperature was maintained at 37 °C. The left pinna was removed and the skull cleared of muscle. The bulla was ventilated to equalize middle ear pressure. The head was held fixed, a left posterior craniotomy made and the cerebellum retracted to expose the CN. A microelectrode assembly was attached to the top of a chamber cemented over the craniotomy and filled with warm mineral oil. Electrodes were advanced into the dorsal surface of the CN, and electrode tracks progressed caudal-rostral through the dorsal, posteroventral, and anteroventral regions. The majority of the responses were recorded from neurons posteroventral and anteroventral portion of the CN. Single-neuron extracellular recordings were made using glass micropipettes filled with 3 M KCl. Action potentials were amplified and discriminated, and the timing was recorded with 1 µs precision. Sound was delivered via a Stax electrostatic phone, calibrated using a ½ inch (B & K 4134) microphone via a probe tube. Signals were generated with 16-bit digital resolution, linearly ramped with a rise-fall time of 3 ms. Signal level was controlled by a 127-dB attenuator with 1-dB steps.

AM signals all had a carrier frequency equal to the characteristic frequency (CF: most sensitive frequency) of the neuron. The modulation frequency (*f*_mod_) was typically varied between 50 and 2,550 Hz in 100-Hz steps, though the maximum varied. The responses of a given neuron where only modulation frequency changes are considered to constitute a single ‘dataset’. Stimuli were 100 ms in duration with a 400 ms interstimulus interval except for certain neurons with pause-buildup response PSTHs (PBU, see [6]). Amplitude-modulated tones were presented at 30, 50, or 70 dB SPL for the carrier frequency and at modulation depths of 50%, 100%, and 200%. Recordings at 200% modulation depth were mainly restricted to the anteroventral CN (Rhode 1994). Most neurons were associated with more than one dataset, varying in sound level or modulation depth. To avoid phase-locking to the carrier, we did not include any neurons with CFs < 3 kHz.

The dataset consisted of 336 units in total, predominantly from the ventral division of the CN. Of these, 191 had been recorded in response to signals with a modulation depth of 100%. Most units were recorded at several sound levels. For some of the analyses, in order to focus on the differences between neurons, we selected out data with 100% modulation depths at the lowest sound level presented (i.e., varying only in modulation frequency), to yield a single modulation transfer function for each neuron.

### Data analysis

#### Pure-tone neuron classification.

Neurons were classified into six physiological types using standard objective methods for the CN [35] in response to 250 repeats of 50 ms pure tones at the CF, 20 and 50 dB above CF threshold. Regular spiking neurons included ChS, ChT and PBU sub-types and irregular spiking neurons were PL and PLN. Onset responses (On) are also associated with diverse responses to tones, cell morphologies, responses to injected current and intrinsic currents [104–106]. However, since our dataset does not contain a sufficient number of onset units (n = 21) to draw meaningful conclusions about subclasses, we will also refer to all onset units as belonging to one class (see [28] for a related and more detailed treatment of mode-locking in onset units).

#### Phase-locking.

The temporal response to amplitude-modulated tones was analyzed from 20 to 100 ms following stimulus onset. Synchronization to modulation frequency was assessed from the PH by calculating the VS [6,24,107,108]:


$$VS=\;\frac{1}{N}\left|\sum_{n=1}^{N}e^{i\varphi_{n}}\right|$$ (1)

where $\begin{array}{c} \varphi \;\; \end{array}$_*n*_ is the phase of the nth spike in the data and N the number of spikes in response to the stimulus. Vector strength analysis of a single dataset (i.e., varying in modulation frequency with all other stimulus conditions remaining constant) yielded a modulation transfer function (MTF-VS).

#### Spike train classification of modulation frequency.

Discrimination between modulation frequencies on the basis of spike timing from individual units was performed using a spike train classifier [36] which has previously been used to study modulation coding in insects. This process is summarized in Fig 1. For a single classifier trial ‘template’ spike trains were drawn randomly from the responses, one for each modulation frequency (*f*_*mod*_). A spike train for classification was then drawn randomly from the remaining set. Spike trains were represented at a binary sequence of 1s and 0s (sample rate: 10 kHz) and convolved with an alpha function,


$$f(t)=t*e^{\frac{-2.45t}{\tau }}\;for\;t>0,\;otherwise\;f(t)\;=\;0$$ (2)

This yielded a time-series function for each spike train, effectively smoothed by the alpha-function, where *τ* is a time constant determining the temporal resolution (smooth gray lines in Fig 1). For a given *τ*, the differences between the convolved template spike trains and the spike train to be classified was computed (red lines in Fig 1), and the resulting time-series functions were squared and summed across time to yield a ‘spike train distance’. Fig 1 shows the distances between every pair of spike trains in a single dataset (for the example neuron in Fig 2). The smallest spike train distance in this set determined the classifier’s decision on that trial. This was repeated until there were 500 trials for every stimulus modulation frequency (with many template combinations). From this, a confusion matrix was generated, where each column describes a probability distribution of classifier decisions for each stimulus modulation frequency.

An unbiased metric of modulation frequency classification, *c′*, was computed from each confusion matrix by fitting a multi-class logistic model [37]. Alternative quantifications of modulation frequency classification can be found in S1 Text. The metric *c′* is related to the probability of a choice via the *softmax* function:


$$\;p\left(chosen\;f_{mod}\;,i|presented\;f_{mod}\;\right)=\frac{\exp\left(c_{i}+B_{i}\right)}{\exp\left(c_{i}+B_{i}\right)+\;\sum_{j\neq i}exp(B_{j})}\;$$ (3)

where *c*_*i*_ quantifies classification performance for the *i*th modulation frequency, and *B*_*j*_ is a bias towards choice *j* irrespective of which modulation frequency is actually presented. In this way, a confusion matrix can be summarized with two vectors: **c**, quantifying the classification of each modulation frequency, and **B** quantifying the biases.

Several characteristics of this model (reviewed in [37]) make it well-suited to comparing across neurons in our dataset. First, it accounts for classification biases toward certain choices, which represent characteristics of the spiking patterns which are not sensitive to changes in modulation frequency and are therefore of little interest here. They can be seen in Figs 2b and 3b as horizontal patterning (see also Fig A in S1 Text). Second, the analysis accounts for the influence of the number of modulation frequencies presented on choice probabilities close to chance, and ceiling effects at high probabilities. The model is conceptually similar to *d’* in an m-alternative forced choice signal-detection model [109–111], hence we refer to this alternative *classification* metric as *c′*, but critically for the model fitting the *softmax* function does not require integration to evaluate.

For each confusion matrix, the vectors **c** and **B** were found by minimizing the sum of squares error of *p (chosen f*_*mod*_*, i|presented f*_*mod*_) in [3] for all *i* and f_mod_. Since *c′* increases exponentially with high values of $p\;(c^{\prime }\;\propto \;-\ln(1-p))$, the confusion matrix probability values were capped at 0.99 before fitting (which in our data cannot be reliably distinguished from *p* = 1), and ***c*** was limited to 8, which corresponds to *p* = 0.9933 for a set of 20 different frequencies (if **B** = 0; note that c′ of 8 corresponds to slightly lower values of p for larger sets of stimuli). In addition, a weak smoothing regularization was imposed, by adding to the computed error a value of 0.0001 times the sum of the squared differences between adjacent values of **c** and **B.** Initial values of **c** were calculated by setting **B** = 0 (for all *j*), and for **B** by setting **c** = 0. **B** was constrained to be positive with the smallest value being fixed at zero, which reduces the number of parameters without loss of any power to fit the model to data.

Each fit was run 10 times with independent uniform noise added to all initial values (±0.0001), generated separately for each fit. Final values of **c** and **B** were averaged across fits. This led to very good fits to the majority of confusion matrices (75% of confusion matrices are fitted with an *R*^2^ of >0.93 and RMS error of <0.02). Representative examples of confusion matrices predicted from fitted parameters are shown in Fig A in S1 Text. Fig A in S1 Text also shows fitted values of **B**, demonstrating how it factors those characteristics of the confusion matrix which are not dependent on which modulation frequency is presented.

Performance was optimized by varying the time constant, *t*, of the alpha function (1, 2, 5, 10, 20, 50 ms). For each dataset (varying only in modulation frequency) in each neuron, the results from the alpha function with the maximum overall *c′* across modulation frequency was taken as the modulation transfer function for *c′* (MTF-*c′*), and used for further analysis.

Classification by ensembles of neurons was tested by an extension of the spike-timing metric to include distance measures from multiple neurons, which has been previously used to estimate decoding accuracy in neural populations [39,112,113]. This metric considers the multiunit spike train distance between two stimulus conditions to be the Pythagorean sum of the individual distances:


$$S_{i,j}=\sqrt{\sum_{n\in N}{{s(n)}_{i,j}}^{2}}\;$$ (4)

where *s*(*n*)_*i,j*_ is the distance between two spike trains *i* and *j*, for neuron *n*. *N* denotes the set of neurons in this particular ensemble.

As with the single neuron calculations, template and test spike trains were drawn at random, but this time for all neurons in the ensemble, and spike overall distances calculated. The classifier chose the template which was closest to the test-spike train ensemble according to equation 4. For each modulation frequency, this process was repeated 500 times to generate a confusion matrix. From the single resulting confusion matrix generated from each ensemble of neurons, c′ was computed in the same way as for single neurons.

This method preserves the identity of each individual neuron, since spike trains are compared within each neuron for any given pair of stimuli, and this result is then combined across neurons. Hence this can be termed a “labeled-line” model. It is distinct from a “summed population” model which combines the responses from different neurons before comparing across stimuli [39] (see [114] for an auditory example).

Equation 4 results in a metric which weights all neurons in the ensemble equally. It is possible to choose these weights to optimize ensemble performance [112]. However, our goal was not to calculate absolute maximum performance (which is in any case likely to be biased by the sample population), but to compare across different neuron types and stimulus conditions. Therefore we opted for the simple scheme of equal weights for all neurons, but varying the number of neurons in an ensemble and in every case choosing those neurons which had the best average individual performance (for *f*_mod_ ≤ 600 Hz) given the ensemble size (e.g., for an ensemble size of 5, we chose the 5 best-performing neurons out of available data). Any given ensemble contained only neurons of one type, with stimuli at a given sound level. Modulation depth was always 100%, and neuron characteristic frequency and the carrier frequency were always greater than 3kHz (mean:12.1 kHz; s.d.:6.3 kHz; 88% above 5 kHz). Characteristic and carrier frequencies could vary in an ensemble, under the assumption this would have little impact on coding since there is little phase-locking to the carrier or component frequencies above 2 kHz in these data [6]. We did not find any systematic differences in classification with characteristic frequency, which pooling would tend to average the effects of. The results of this analysis proved straightforward to interpret.

#### Simulating “control” neurons which are well characterized using Vector Strength.

This spike train classification method described is sensitive to any differences in absolute spike timing across different stimulus conditions, and it is sensitive to the reliability of spike timing from trial-to-trial (illustrated in Fig 1). This contrasts with the VS measure which summarizes the tendency for spikes to be close to the mean phase of firing as a single value. To quantify the added value of additional information in the spike train over this mean phase preference, we also simulated a “control” neuron which was well characterized by the VS value alone.

Simulated spike trains were generated using the von-Mises (“circular normal”) distribution which is a reasonable summary of PHs of cat auditory nerve fibers [115]. For a modulation period *T*_mod_ = 1/*f*_mod_, the density function is:


$$p_{\kappa ,f_{mod}\;(t)=\;\frac{1}{T\;I_{o}(\kappa )}\;exp\left(\kappa \;cos\left(2\pi f_{mod}\;t-\;\mu \;\right)\right)\;}$$ (5)

where *m* is the mean phase and *k* is the concentration parameter, which is large for high values of VS where spikes are concentrated close to the mean phase, and $\begin{array}{c} I_{o}(\kappa )=\;\frac{1}{2\pi }\int_{-\pi }^{\pi }exp\left(\kappa \cos\left(x\right)\right)dx \end{array}$.

Individual spike trains were simulated as an inhomogeneous Poisson process with a periodic time-varying firing rate which followed the von-Mises distribution across the modulation cycle such that the VS and mean spike rates matched the responses of individual neurons in response to individual stimuli [116]:


$$\lambda (t)=T\overset{―}{\;\lambda \;}p_{\kappa ,f_{mod}}(t)$$ (6)

where $\overset{―}{\;\lambda \;}$ is the mean firing rate of the neuron, and *k* and *m* (in Equation 5) are set to match the VS and mean phase of that neuron in that stimulus condition.

The simulated conditions mimicked the conditions run for the neuron, and the simulated spike trains were subjected to the same classification process. The resulting *c′* (Figs 2c and 3c) represents the classifier performance that would be expected if the measured VS provided a complete characterization of the neuron’s behavior in response to amplitude modulation.

#### Statistical models of modulation classification across the entire data set.

To explore how modulation classification was related to the responses of individual neurons, different neuron types, and stimulus conditions, we developed several simple statistical models that related *c′* to a combination of stimulus conditions and the statistics of the neuron’s responses. The same method was also applied to classifier hit-rate and F1 score (see S1 Text).

First, we derived a best-fitting population modulation transfer function *shape*, Φ(*f*_*mod*_), optimized across the entire dataset. This function is related to each individual MTF-*c′* function by only a single scale factor:


$$c^{\prime }(predicted)=\;\Phi \left(f_{mod}\right)S_{i}$$ (7)

where *S*_*i*_ is the individual scaling factor which best predicts the *i*th MTF-c′ in the data. Values of Φ(*f*_*mod*_) are chosen for each modulation frequency to minimize sum of square error with all values of *c′* across the entire dataset, with values of *S*_*i*_ being treated as random effects. This empirically derived function was normalized after fitting to be 1 at 50 Hz, and the resulting function values dropped to ~0.1 by 1 kHz (shown in Fig 5c). This yields a model which is the theoretical best fit of a single transfer function *shape* to the data.

A different form of this model, which sought to determine the underlying factors which determine the differences between different neurons, and datasets within each neuron, replaced *S*_*i*_ with a function of sound level and modulation depth. Considering first how the stimulus conditions alone predict neural envelope classification:


$$c^{\prime }(predicted)=\;\Phi \left(f_{mod}\right)\left[l_{1}\lambda_{30}+l_{2}\lambda_{50}+d_{1}\delta_{1}+d_{2}\delta_{2}\;\right]$$ (8)

Sound level and modulation depth were represented as 3-level categorical variables. Sound levels were 30, 50, and 70 dB SPL, with other levels (which constituted 30% of the entire dataset) rounded to these values in order to reduce the number of model parameters. Modulation depths were 50%, 100%, and 200%, respectively (other shallower modulation depths constituted only 2% of the entire dataset and are omitted from analysis). Thus, *l*_1_*, l*_2_*, d*_1_, and *d*_2_ are free parameters in the model, and *λ* and δ code levels and depths (i.e., *λ*_*30*_ = 1 when the sound level is 30 dB SPL and is zero otherwise, enabling *l*_1,2_ and *d*_1,2_ to behave as would category levels in an ANOVA, with *n −* 1 = 2 degrees of freedom. Thus, parameters in Equation 8 measure the departure from the mean values for 70 dB SPL and 100% modulation depth). The result was a 4-parameter model fitted to 916 MTF-*c′* functions.

In order to explore how neuron type contributed to classification, above that of the stimulus condition, a third form of this model included an additional term, *σ*_*i*_ representing the neuron type.


$$c^{\prime }(predicted)=\;\Phi \left(f_{mod}\right)\left[l_{1}\lambda_{30}+l_{2}\lambda_{50}+d_{1}\delta_{1}+d_{2}\delta_{2}+\sum iw_{i}\sigma_{i}\;\right]$$ (9)

and *w*_*i*_ was a scaling factor for neuron type, chosen to maximize the fit to all of the data.

To explore how specific aspects of spike timing contributed to classification, above that of the stimulus condition, a final form of this model included one or more additional terms, each of which were based on a spike train statistic (e.g., VS):


$$c^{\prime }(predicted)=\;\Phi \left(f_{mod}\right)\left[l_{1}\lambda_{30}+l_{2}\lambda_{50}+d_{1}\delta_{1}+d_{2}\delta_{2}+w\sigma_{i}+\ldots \;\right]$$ (10)

where *σ*_*I*_ could be some characteristic of the neuron or individual modulation transfer function (e.g., VS, Z_ISI_ (described below), CV) and *w* was a single weighting factor chosen to maximize the fit to all the data.

#### Spike-timing dependence: Z_ISI_ metric.

We derived several statistics to describe the way that information was encoded in spike trains, which were included in the non-linear regression models as predictors. These statistics indexed conceptually (if not mathematically) distinct aspects of the spike code.

A simple model of spiking behavior in a neuron is that consecutive spikes occur independently as a simple probabilistic function of the stimulus. The precise timing of spikes can carry information about the stimulus, encoded as a rapidly varying firing rate. In the case here, where the stimulus is varying periodically, the PH provides a complete description of the spiking behavior as the varying rate across one period.

To test whether the phase of the stimulus at which the spike occurred could account for ISI statistics (for full details see: [28], see also: [117]), a surrogate spike train was created for each original spike train by randomly shuffling the period within which each spike occurred, whilst maintaining the phase of each spike. “Shuffled” ISI distributions should be the same as the originals if the phase alone completely described the spike timing behavior. For each pair of spike trains *i,j* (*i* ≠ *j*) the difference between the original and shuffled ISI distributions (*x*_*ij*_) was calculated as the root mean square error (RMSE) between the ISI probability density functions of the original spike train *i* and shuffled spike train *j*. The RMSE was also computed between both original spike trains *i* and *j* (*y*_*ij*_). A *z*-score statistic was then computed as (*E*{*x*} − *E*{y})/(*E*{y^2^} − *E*{y}^2^)^1/2^. This calculation was repeated for 100 sets of surrogate spike trains and averaged to give a final Z_ISI_ statistic. In effect, the Z_ISI_ metric characterizes the degree to which the timing of the next spike depends on the previous spike, and not on the phase of the modulation, as expected for pure Poisson or firing rate behavior.

Inclusion of the statistics in the regression models was performed in one of two ways. In most instances, we took a single value of Z_ISI_ from each modulation transfer function, computed for the modulation frequency closest to 150 Hz. This choice was not critical, since within a single modulation transfer function, Z_ISI_ was correlated across modulation frequency, and dropped with increasing modulation frequency. In the second approach (explored in S1 Text) Z_ISI_, as well as other statistics, were taken from each individual modulation frequency and used within the regression models.

#### Inter-spike interval statistics to characterize temporal structure: Shuffled autocorrelation.

Inter-spike interval statistics were computed from normalized shuffled auto-correlograms [SACs: 118]. To characterize the peaks in the SACs we employed a peak-picking algorithm to pick out the locations of the statistically significant peaks in the SAC in the range  ±1.1 × the modulation period. The algorithm iteratively sub-divided the SAC into progressively more local minima and maxima. Specifically, starting with the whole function, the maximum and minimum in the function were found. The maximum determined a peak in the SAC. The SAC function was then subdivided at the point of the found maximum, and the maxima and minima in each subdivision found, to potentially identify two more peaks. Peaks were identified, and sub-division continued, as long as the distance from peaks to adjacent troughs exceeded the peak-to-trough distance measured in the SACs of random spike trains of the same duration and spike count, with *p* < 0.001 (the bootstrapped probability of observing a given peak-to-trough distance). Visual inspection of the output revealed that this was successful in identifying and quantifying peaks at low modulation frequencies.

The peak-to-trough distances of peaks within the SAC were used as statistics within the regression models. Each of the peaks became a separate predictor variable in the model. SAC functions are near symmetrical around an autocorrelation lag of zero, and form repetitive decaying patterns which repeat at multiples of the lag corresponding to the period of the modulation. Therefore, we only included the zero-lag peak, the peak at a positive lag of one period, and (up to) the four largest peaks between these. Non-significant peaks were coded as having an amplitude of zero. Similar to Z_ISI_, these values were taken either from a single modulation frequency for each modulation transfer function, or individually from each modulation frequency. Following inspection of the pair-wise correlations between the heights of different peaks within the SAC, and a systematic exploration of the inclusion of different peaks within the regression models, the zero-lag peak was omitted from most presented models as it was highly correlated (*R*^2^ = 0.81) with the peak at the lag corresponding to the modulation period and including both did not improve the predictive power of models.

#### PSTH derived statistics: Reliability and neural fluctuation.

From the peristimulus time histograms (PSTHs), we derived two statistics. *Reliability* [41] is a way of measuring the variability in response across trials. This was computed as the Pearson correlation of two separate PSTHs derived from the responses to the odd and even presentations of a given stimulus. Prior to computing the Pearson correlation, each PSTH was convolved with a Gaussian smoothing window with a standard deviation of 0.24 ms. *Neural fluctuation* [41] measures the total amount of change in the firing rate throughout the response, as an index of the magnitude of the temporal features, which does not require them to be related to the stimulus or any particular coding strategy. *Neural fluctuation* was computed from the PSTH made from all presentations of a given stimulus. The PSTH was first convolved with a Gaussian window having a standard deviation of 0.48 ms before the *neural fluctuation* was computed as the sum of the absolute value of the first differential, normalized by dividing by the length of the PSTH. Similar to Z_ISI_, these statistics were taken either from a single modulation frequency of 150 Hz for each modulation transfer function, or individually from each modulation frequency.

## Supporting information

S1 Text **Fig A. Examples of fitted confusion matrices using the *softmax* model. A.** The example sustained chopper neuron from Fig 2 in the paper. **b.** The example primary-like neuron from Fig 3 in the paper. **c.** A primary-like notch neuron which demonstrates a clear where the classifier is systematically bias toward choosing high modulation frequencies. **d.** An onset-chopper neuron which is less well modeled than 88% of the data. The leftmost panels reproduce the directly calculated confusion matrix. The middle panel shows the predicted confusion matrix from the best fitting values of *c′* and *B*. The rightmost panel shows *c′* and *B*. Dotted lines show the maximum and minimum values derived from 10 separate fits run with different starting parameters, and give some indication of the reliability of the fitting process. The orange line shows the hit rate for comparison. **Fig B. Modulation Transfer Functions as a function of sound level when modulation depth is 200%, derived from a spike timing classifier (MTF-c*′*) or phase-locking (MTF-VS). a.** Maximum c*′* at the peak of the corresponding modulation transfer function, split by neuron type, and sound level. **b.** Proportion of MTF-c*′* shapes classified as lowpass, split by neuron type and sound level. **c.** Mean population MTF-c*′* for sustained chopper neurons as a function of sound level, frequency-normalized to BMD-c*′*. **d.** Mean population MTF-c*′* for primary-like neurons as a function of sound level. **e.** Peak vector strength values and **f**. proportion of lowpass MTF-VS as a function of neuron type and level. **g.** Mean population MTF-VS for sustained chopper neurons as a function of sound level, frequency normalized to BMF-VS. **h.** Mean population MTF-VS for primary-like neurons as a function of sound level. **Fig C. Classifier Modulation Transfer Functions (MTF-*c′*) at shallow modulation depths is 200%. a.** Modulation transfer functions in sustained chopper neurons for a modulation depth of 50%. Individual functions where *c′* >1 for at least one modulation frequency are shown as colored lines (*n* = 12). Functions which do not exceed *c′* = 1 are shown in gray (*n* = 7). **b.** Modulation transfer functions in sustained chopper neurons for a modulation depth of 20%. **c.** Modulation transfer functions in all other neuron types for a modulation depth of 50%. **d.** Modulation transfer functions in all other neuron types for a modulation depth of 20%. **Fig D. Classifier Modulation Transfer Functions (MTF-*c′*) for small differences in modulation frequency. a.** Modulation transfer functions in seven sustained chopper neurons and one pause/build-up neuron for a modulation frequency steps of 25 Hz. Line style represents sound level. Different lines with the same style are different example neurons. **b.** Modulation transfer functions in a single transient chopper-neuron at very low modulation frequencies (<50 Hz) and very small frequency steps (5 Hz) at several sound levels. **Fig E. Details of the behavior of the Z**_**ISI**_ **statistic. a.** The frequency dependence of Z_ISI_ for each neuron type. Shaded areas show standard error of the mean. **b.** Mean values of Z_ISI_ from each dataset, calculated as the average of all values for f_mod_ < 1kHz. Dashed line indicates a value of 2 (units are standard deviation) which was proposed by Laudanski and colleagues (2010) as one of the criteria for mode-locking. **c.** Mean values of Vector Strength for each dataset (mean for *f*_mod_ <1 kHz), following the interval shuffling proposed as the second criteria for mode-locking. **d.** Rayleigh criterion values following interval shuffling. Laudanski and colleagues proposed phase locking should be nonsignificant following interval shuffling if responses were mode-locked. Dashed line indicates a Rayleigh value of 13.8 (below this corresponding to *p* > 0.001). **Fig F. Further comparisons of how different statistics predict classification performance.** In most cases, the statistics are drawn from *f*_mod_ ~ 150 Hz and applied across all modulation frequencies within a dataset, and the stated number of parameters is added to a model with stimulus-only parameters. Purple bars show the predictive power when the statistics are instead drawn from the specific modulation frequencies. Note that the theoretical maximum does not apply to the purple bars, since the predictions can vary in frequency dependence (transfer function shape) between datasets. The underlying data for this figure is contained in S4 Data. **Fig G. Frequency dependence of the number of significant SAC peaks for each neuron type. The underlying data for this figure is contained in** S5 Data**. Fig H. How different SAC peaks contribute to predictions of classification performance.** In all cases, the statistic is drawn from *f*_mod_ ~ 150 Hz and applied across all modulation frequencies within a dataset, and the stated number of parameters is added to a model with stimulus-only parameters. The underlying data for this figure is contained in S6 Data. **Fig I. Reproducing the main findings using classifier hit-rate**. **a.** Examples of classifier performance as hit rate, for the exact same sets of neurons as in Fig 4. **b.** The range best modulation frequencies when classification is expressed as hit rate, for each neuron type at low-level sound levels. This reproduces Fig 6a. **c.** Mean of modulation transfer functions relative to hit rate BMF, at low sound levels. This reproduces Fig 6b. **d.** Mean of modulation transfer functions for each neuron type as a function of absolute modulation frequency, reproducing Fig 5a. **e.** The best possible prediction of hit rate when the transfer function is modeled as a single function which can only differ by a scale factor between neurons, sound levels and modulation depth. **f.** Reproduction of the statistical regression models from Fig 9a, when classifier performance is expressed as hit rate. **g.** Modulation transfer functions for small populations of neurons when hit rate is the measure of classifier performance. The underlying data for this figure is contained in S7 Data. **Fig J. Reproducing the main findings using classifier F1-score. a.** Examples of classifier performance as F1-score, for the exact same sets of neurons as in Fig 4. **b.** The range best modulation frequencies when classification is expressed as F1 score, for each neuron type at low-level sound levels. This reproduces Fig 6a. **c.** Mean of modulation transfer functions relative to F1-score BMF, at low sound levels. This reproduces Fig 6b. **d.** Mean of F1 modulation transfer functions for each neuron type as a function of absolute modulation frequency, reproducing Fig 5a. **e.** The best possible prediction of F1-score when the transfer function is modeled as a single function which can only differ by a scale factor between neurons, sound levels, and modulation depth. **f.** Reproduction of the statistical regression models from Fig 9a, when classifier performance is expressed as F1-score. **g.** Modulation transfer functions for small populations of neurons when F1-score is the measure of classifier performance. The underlying data for this figure is contained in S8 Data. **Fig K. Pairwise correlations between the various predictors in the statistical regression**. The statistics apply to responses for *f*_mod_ ~ 150 Hz. To reduce the total number of plots, the SAC peaks are shown as a simple unweighted sum. **Fig L. The classifier resolutions which yield the best performance, split by neuron type. The underlying data for this figure is contained in** S9 Data**. Fig M. Partial reproduction of Fig 6 in the main paper showing the impact of a reduced temporal resolution in the classifier.** Upper panels **(a)** and **(b)** show results reproduce these panels as they appear in the main paper, where the classifier resolution was chosen to maximize the performance of each MTF. Lower panels **(c)** and **(d)** show the equivalent analysis for a fixed classifier resolution of *τ* = 10 ms. The underlying data for this figure is contained in S10 Data. **Fig N. Reproduction of Fig 7 in the main paper showing the impact of a reduced temporal resolution in the classifier.** Upper panels **(a)–(d)** reproduce these panels as they appear in the submitted manuscript, where the classifier resolution was chosen to maximize the performance of each MTF. Lower panels **(e)–(h)** show the same analysis for a fixed classifier resolution of *τ* = 10 ms.
(PDF)

S1 Data The individual numerical values that underlie the summary data displayed in Fig 5b, 6a, and 6c. (XLSX)

S2 Data The individual numerical values that underlie the summary data displayed in Fig 9a. (XLSX)

S3 Data The individual numerical values that underlie the summary data displayed in Fig 9b, 9c, 9e, and 9f. (XLSX)

S4 Data The individual numerical values that underlie the summary data displayed in Fig F in S1 Text. (XLSX)

S5 Data The individual numerical values that underlie the summary data displayed in Fig G in S1 Text. (XLSX)

S6 Data The individual numerical values that underlie the summary data displayed in Fig H in S1 Text. (XLSX)

S7 Data The individual numerical values that underlie the summary data displayed in Fig I in S1 Text. (XLSX)

S8 Data The individual numerical values that underlie the summary data displayed in Fig J in S1 Text. (XLSX)

S9 Data The individual numerical values that underlie the summary data displayed in Fig L in S1 Text. (XLSX)

S10 Data The individual numerical values that underlie the summary data displayed in Fig M in S1 Text. (XLSX)

## Abbreviations

AM: amplitude-modulated
BMF: Best Modulation Frequency
ChS: sustained chopper
ChT: transient chopper
CN: cochlear nucleus
CV: coefficient of variation
IC: inferior colliculus
ISI: interspike interval
ITDs: interaural timing delays
LSO: lateral superior olivary nucleus
MNTB: medial nucleus of the trapezoid body
PBU: pause/build-up
PH: period histogram
PL: primary-like
PLN: primary-like notch
PSTHs: peristimulus time histograms
RMSE: root mean square error
VS: vector strength

## References

[1] Shannon RV, Zeng FG, Kamath V, Wygonski J, Ekelid M. Speech recognition with primarily temporal cues. Science. 1995;270(5234):303–4. doi: 10.1126/science.270.5234.303 7569981

[2] Dorman MF, Loizou PC, Rainey D. Speech intelligibility as a function of the number of channels of stimulation for signal processors using sine-wave and noise-band outputs. J Acoust Soc Am. 1997;102(4):2403–11. doi: 10.1121/1.419603 9348698

[3] Singh NC, Theunissen FE. Modulation spectra of natural sounds and ethological theories of auditory processing. J Acoust Soc Am. 2003;114(6 Pt 1):3394–411. doi: 10.1121/1.1624067 14714819

[4] Joris PX, Yin TC. Responses to amplitude-modulated tones in the auditory nerve of the cat. J Acoust Soc Am. 1992;91(1):215–32. doi: 10.1121/1.402757 1737873

[5] Frisina RD, Smith RL, Chamberlain SC. Encoding of amplitude modulation in the gerbil cochlear nucleus: I. A hierarchy of enhancement. Hear Res. 1990;44(2–3):99–122. doi: 10.1016/0378-5955(90)90074-y 2329098

[6] Rhode WS, Greenberg S. Encoding of amplitude modulation in the cochlear nucleus of the cat. J Neurophysiol. 1994;71(5):1797–825. 8064349 10.1152/jn.1994.71.5.1797

[7] Mackevicius EL, Best MD, Saal HP, Bensmaia SJ. Millisecond precision spike timing shapes tactile perception. J Neurosci. 2012;32(44):15309–17. doi: 10.1523/JNEUROSCI.2161-12.2012 23115169 PMC3752122

[8] Foffani G, Tutunculer B, Moxon KA. Role of spike timing in the forelimb somatosensory cortex of the rat. J Neurosci. 2004;24(33):7266–71. doi: 10.1523/JNEUROSCI.2523-04.2004 15317852 PMC6729767

[9] Engineer CT, Perez CA, Chen YH, Carraway RS, Reed AC, Shetake JA, et al. Cortical activity patterns predict speech discrimination ability. Nat Neurosci. 2008;11(5):603–8. doi: 10.1038/nn.2109 18425123 PMC2951886

[10] Akbari H, Khalighinejad B, Herrero JL, Mehta AD, Mesgarani N. Towards reconstructing intelligible speech from the human auditory cortex. Sci Rep. 2019;9(1):874. doi: 10.1038/s41598-018-37359-z 30696881 PMC6351601

[11] Joris PX, Schreiner CE, Rees A. Neural processing of amplitude-modulated sounds. Physiol Rev. 2004;84(2):541–77. doi: 10.1152/physrev.00029.2003 15044682

[12] Vázquez Y, Salinas E, Romo R. Transformation of the neural code for tactile detection from thalamus to cortex. Proc Natl Acad Sci U S A. 2013;110(28):E2635-44. doi: 10.1073/pnas.1309728110 23798408 PMC3710840

[13] Young ED, Oertel D. Cochlear nucleus. In: Shepherd GM, editor. The synaptic organization of the brain, vol. 4. 5th ed. Oxford: Oxford University Press; 2004. p. 125–63.

[14] Sayles M, Fullgrabe C, Winter IM. Neurometric amplitude-modulation detection threshold in the guinea-pig ventral cochlear nucleus. J Physiol. 2013;591(Pt 13):3401–19. 23629508 10.1113/jphysiol.2013.253062 PMC3717235

[15] Rees A, Palmer AR. Neuronal responses to amplitude-modulated and pure-tone stimuli in the guinea pig inferior colliculus, and their modification by broadband noise. J Acoust Soc Am. 1989;85(5):1978–94. doi: 10.1121/1.397851 2732379

[16] Souffi S, Lorenzi C, Varnet L, Huetz C, Edeline JM. Noise-sensitive but more precise subcortical representations coexist with robust cortical encoding of natural vocalizations. J Neurosci. 2020;40(27):5228–46. 32444386 10.1523/JNEUROSCI.2731-19.2020 PMC7329308

[17] Voss RF, Clarke J. ‘1/fnoise’ in music and speech. Nature. 1975;258(5533):317–8. doi: 10.1038/258317a0

[18] Nelson PC, Carney LH. Neural rate and timing cues for detection and discrimination of amplitude-modulated tones in the awake rabbit inferior colliculus. J Neurophysiol. 2007;97(1):522–39. doi: 10.1152/jn.00776.2006 17079342 PMC2577033

[19] Winter IM, Palmer AR. Level dependence of cochlear nucleus onset unit responses and facilitation by second tones or broadband noise. J Neurophysiol. 1995;73(1):141–59. doi: 10.1152/jn.1995.73.1.141 7714560

[20] Viemeister NF. Temporal modulation transfer functions based upon modulation thresholds. J Acoust Soc Am. 1979;66(5):1364–80. doi: 10.1121/1.383531 500975

[21] Kohlrausch A. Comment on “Temporal modulation transfer functions in patients with cochlear implants” [J. Acoust. Soc. Am. 91,2156-2164 (1992)]. J Acoust Soc Am. 1993;93(3):1649–52. 8473614 10.1121/1.406826

[22] Kohlrausch A, Fassel R, Dau T. The influence of carrier level and frequency on modulation and beat-detection thresholds for sinusoidal carriers. J Acoust Soc Am. 2000;108(2):723–34. doi: 10.1121/1.429605 10955639

[23] Wojtczak M. The effect of carrier level on tuning in amplitude-modulation masking. J Acoust Soc Am. 2011;130(6):3916–25. doi: 10.1121/1.3658475 22225047 PMC3253595

[24] Goldberg JM, Brown PB. Response of binaural neurons of dog superior olivary complex to dichotic tonal stimuli: some physiological mechanisms of sound localization. J Neurophysiol. 1969;32(4):613–36. doi: 10.1152/jn.1969.32.4.613 5810617

[25] Keener JP, Hoppensteadt FC, Rinzel J. Integrate-and-fire models of nerve membrane response to oscillatory input. SIAM J Appl Math. 1981;41(3):503–17. doi: 10.1137/0141042

[26] Coombes S, Bressloff PC. Mode locking and Arnold tongues in integrate-and-fire neural oscillators. Phys Rev E Stat Phys Plasmas Fluids Relat Interdiscip Topics. 1999;60(2 Pt B):2086–96. doi: 10.1103/physreve.60.2086 11970001

[27] Mainen ZF, Sejnowski TJ. Reliability of spike timing in neocortical neurons. Science. 1995;268(5216):1503–6. doi: 10.1126/science.7770778 7770778

[28] Laudanski J, Coombes S, Palmer AR, Sumner CJ. Mode-locked spike trains in responses of ventral cochlear nucleus chopper and onset neurons to periodic stimuli. J Neurophysiol. 2010;103(3):1226–37. doi: 10.1152/jn.00070.2009 20042702 PMC2887620

[29] Rhode WS, Smith PH. Encoding timing and intensity in the ventral cochlear nucleus of the cat. J Neurophysiol. 1986;56(2):261–86. doi: 10.1152/jn.1986.56.2.261 3760921

[30] Wei L, Karino S, Verschooten E, Joris PX. Enhancement of phase-locking in rodents. I. An axonal recording study in gerbil. J Neurophysiol. 2017;118(4):2009–23. doi: 10.1152/jn.00194.2016 28701535 PMC5626893

[31] Rhode WS. Temporal coding of 200% amplitude modulated signals in the ventral cochlear nucleus of cat. Hear Res. 1994;77(1–2):43–68. doi: 10.1016/0378-5955(94)90252-6 7928738

[32] Young ED, Robert JM, Shofner WP. Regularity and latency of units in ventral cochlear nucleus: implications for unit classification and generation of response properties. J Neurophysiol. 1988;60(1):1–29. doi: 10.1152/jn.1988.60.1.1 3404211

[33] Pfeiffer RR. Classification of response patterns of spike discharges for units in cochlear nucleus—tone-burst stimulation. Exp Brain Res. 1966;1(3):220. 5920550 10.1007/BF00234343

[34] Godfrey DA, Kiang NY, Norris BE. Single unit activity in the posteroventral cochlear nucleus of the cat. J Comp Neurol. 1975;162(2):247–68. 1150921 10.1002/cne.901620206

[35] Blackburn CC, Sachs MB. Classification of unit types in the anteroventral cochlear nucleus: PST histograms and regularity analysis. J Neurophysiol. 1989;62(6):1303–29. doi: 10.1152/jn.1989.62.6.1303 2600627

[36] Wohlgemuth S, Ronacher B. Auditory discrimination of amplitude modulations based on metric distances of spike trains. J Neurophysiol. 2007;97(4):3082–92. doi: 10.1152/jn.01235.2006 17314239

[37] Carandini M. Sensory choices as logistic classification. Neuron. 2024;112(17):2854-2868.e1. doi: 10.1016/j.neuron.2024.06.016 39013468 PMC11377159

[38] Rees A, Moller AR. Stimulus properties influencing the responses of inferior colliculus neurons to amplitude-modulated sounds. Hear Res. 1987;27(2):129–43. 3610842 10.1016/0378-5955(87)90014-1

[39] Aronov D, Reich DS, Mechler F, Victor JD. Neural coding of spatial phase in V1 of the macaque monkey. J Neurophysiol. 2003;89(6):3304–27. doi: 10.1152/jn.00826.2002 12612048

[40] Laudanski J, Sumner C, Coombes S. Calcium window currents, periodic forcing, and chaos: understanding single neuron response with a discontinuous one-dimensional map. Phys Rev E Stat Nonlin Soft Matter Phys. 2010;82(1 Pt 1):011924. doi: 10.1103/PhysRevE.82.011924 20866665

[41] Gai Y, Carney LH. Temporal measures and neural strategies for detection of tones in noise based on responses in anteroventral cochlear nucleus. J Neurophysiol. 2006;96(5):2451–64. doi: 10.1152/jn.00471.2006 16914617 PMC2577022

[42] Lee J. Amplitude modulation rate discrimination with sinusoidal carriers. J Acoust Soc Am. 1994;96(4):2140–7. doi: 10.1121/1.410156 7963027

[43] Cooper NP, Guinan JJ Jr. Efferent-mediated control of basilar membrane motion. J Physiol. 2006;576(Pt 1):49–54. doi: 10.1113/jphysiol.2006.114991 16901947 PMC1995651

[44] Hockley A, Wu C, Shore SE. Olivocochlear projections contribute to superior intensity coding in cochlear nucleus small cells. J Physiol. 2022;600(1):61–73. doi: 10.1113/JP282262 34761815 PMC9067393

[45] Gosselin E, Bagur S, Bathellier B. Massive perturbation of sound representations by anesthesia in the auditory brainstem. Sci Adv. 2024;10(42):eado2291. doi: 10.1126/sciadv.ado2291 39423272 PMC11488538

[46] Sumner CJ, Wells TT, Bergevin C, Sollini J, Kreft HA, Palmer AR, et al. Mammalian behavior and physiology converge to confirm sharper cochlear tuning in humans. Proc Natl Acad Sci U S A. 2018;115(44):11322–6. doi: 10.1073/pnas.1810766115 30322908 PMC6217411

[47] Verschooten E, Desloovere C, Joris PX. High-resolution frequency tuning but not temporal coding in the human cochlea. PLoS Biol. 2018;16(10):e2005164. doi: 10.1371/journal.pbio.2005164 30321166 PMC6201958

[48] Manis PB, Campagnola L. A biophysical modelling platform of the cochlear nucleus and other auditory circuits: from channels to networks. Hear Res. 2018;360:76–91. doi: 10.1016/j.heares.2017.12.017 29331233 PMC6053909

[49] Zilany MSA, Bruce IC, Carney LH. Updated parameters and expanded simulation options for a model of the auditory periphery. J Acoust Soc Am. 2014;135(1):283–6. doi: 10.1121/1.4837815 24437768 PMC3985897

[50] Smalt CJ, Heinz MG, Strickland EA. Modeling the time-varying and level-dependent effects of the medial olivocochlear reflex in auditory nerve responses. J Assoc Res Otolaryngol. 2014;15(2):159–73. 24306278 10.1007/s10162-013-0430-z PMC3946143

[51] Grange J, Zhang 张 梦 超 M, Culling J. The role of efferent reflexes in the efficient encoding of speech by the auditory nerve. J Neurosci. 2022;42(36):6907–16. 35882559 10.1523/JNEUROSCI.2220-21.2022 PMC9463981

[52] van Rossum MC. A novel spike distance. Neural Comput. 2001;13(4):751–63. doi: 10.1162/089976601300014321 11255567

[53] Victor JD, Purpura KP. Nature and precision of temporal coding in visual cortex: a metric-space analysis. J Neurophysiol. 1996;76(2):1310–26. doi: 10.1152/jn.1996.76.2.1310 8871238

[54] Chase SM, Young ED. Spike-timing codes enhance the representation of multiple simultaneous sound-localization cues in the inferior colliculus. J Neurosci. 2006;26(15):3889–98. doi: 10.1523/JNEUROSCI.4986-05.2006 16611804 PMC6673902

[55] Rhode WS. Response patterns to sound associated with labeled globular/bushy cells in cat. Neuroscience. 2008;154(1):87–98. 18423882 10.1016/j.neuroscience.2008.03.013 PMC2518325

[56] Rothman JS, Manis PB. Differential expression of three distinct potassium currents in the ventral cochlear nucleus. J Neurophysiol. 2003;89(6):3070–82. doi: 10.1152/jn.00125.2002 12783951

[57] Paolini AG, Clarey JC, Needham K, Clark GM. Fast inhibition alters first spike timing in auditory brainstem neurons. J Neurophysiol. 2004;92(4):2615–21. 15140909 10.1152/jn.00327.2004

[58] Bernstein LR, Trahiotis C. Detection of interaural delay in high-frequency sinusoidally amplitude-modulated tones, two-tone complexes, and bands of noise. J Acoust Soc Am. 1994;95(6):3561–7. doi: 10.1121/1.409973 8046145

[59] Joris PX, Yin TC. Envelope coding in the lateral superior olive. I. Sensitivity to interaural time differences. J Neurophysiol. 1995;73(3):1043–62. doi: 10.1152/jn.1995.73.3.1043 7608754

[60] Spangler KM, Warr WB, Henkel CK. The projections of principal cells of the medial nucleus of the trapezoid body in the cat. J Comp Neurol. 1985;238(3):249–62. doi: 10.1002/cne.902380302 4044914

[61] Beiderbeck B, Myoga MH, Müller NIC, Callan AR, Friauf E, Grothe B, et al. Precisely timed inhibition facilitates action potential firing for spatial coding in the auditory brainstem. Nat Commun. 2018;9(1):1771. doi: 10.1038/s41467-018-04210-y 29720589 PMC5932051

[62] Franken TP, Joris PX, Smith PH. Principal cells of the brainstem’s interaural sound level detector are temporal differentiators rather than integrators. Elife. 2018;7:e33854. doi: 10.7554/eLife.33854 29901438 PMC6063729

[63] Greenberg D, Monaghan JJM, Dietz M, Marquardt T, McAlpine D. Influence of envelope waveform on ITD sensitivity of neurons in the auditory midbrain. J Neurophysiol. 2017;118(4):2358–70. doi: 10.1152/jn.01048.2015 28701550 PMC5646199

[64] Bernstein LR, Trahiotis C. Sensitivity to envelope-based interaural delays at high frequencies: center frequency affects the envelope rate-limitation. J Acoust Soc Am. 2014;135(2):808–16. doi: 10.1121/1.4861251 25234889 PMC3985968

[65] Batra R, Kuwada S, Stanford TR. Temporal coding of envelopes and their interaural delays in the inferior colliculus of the unanesthetized rabbit. J Neurophysiol. 1989;61(2):257–68. doi: 10.1152/jn.1989.61.2.257 2918354

[66] Monaghan JJM, Bleeck S, McAlpine D. Sensitivity to envelope interaural time differences at high modulation rates. Trends Hear. 2015;19:2331216515619331. doi: 10.1177/2331216515619331 26721926 PMC4871209

[67] Joris PX, Smith PH. The volley theory and the spherical cell puzzle. Neuroscience. 2008;154(1):65–76. doi: 10.1016/j.neuroscience.2008.03.002 18424004 PMC2486254

[68] Ashida G, Kretzberg J, Tollin DJ. Roles for coincidence detection in coding amplitude-modulated sounds. PLoS Comput Biol. 2016;12(6):e1004997. doi: 10.1371/journal.pcbi.1004997 27322612 PMC4920552

[69] Hewitt MJ, Meddis R. A computer model of amplitude-modulation sensitivity of single units in the inferior colliculus. J Acoust Soc Am. 1994;95(4):2145–59. doi: 10.1121/1.408676 8201111

[70] Bahmer A, Langner G. Oscillating neurons in the cochlear nucleus: II. Simulation results. Biol Cybern. 2006;95(4):381–92. 16847667 10.1007/s00422-006-0091-7

[71] Langner G, Schreiner CE. Periodicity coding in the inferior colliculus of the cat. I. Neuronal mechanisms. J Neurophysiol. 1988;60(6):1799–822. doi: 10.1152/jn.1988.60.6.1799 3236052

[72] Schnupp JWH, Garcia-Lazaro JA, Lesica NA. Periodotopy in the gerbil inferior colliculus: local clustering rather than a gradient map. Front Neural Circuits. 2015;9:37. doi: 10.3389/fncir.2015.00037 26379508 PMC4550179

[73] Bendor D, Wang X. Neural coding of periodicity in marmoset auditory cortex. J Neurophysiol. 2010;103(4):1809–22. doi: 10.1152/jn.00281.2009 20147419 PMC2853289

[74] Johnson JS, Niwa M, O’Connor KN, Sutter ML. Amplitude modulation encoding in auditory cortex: comparisons between the primary and middle lateral belt regions. bioRxiv. 2020:2020.03.05.979575. 10.1152/jn.00171.2020 PMC7814894 33026929

[75] Krishna BS, Semple MN. Auditory temporal processing: responses to sinusoidally amplitude-modulated tones in the inferior colliculus. J Neurophysiol. 2000;84(1):255–73. doi: 10.1152/jn.2000.84.1.255 10899201

[76] Kim DO, Carney L, Kuwada S. Amplitude modulation transfer functions reveal opposing populations within both the inferior colliculus and medial geniculate body. J Neurophysiol. 2020;124(4):1198–215. doi: 10.1152/jn.00279.2020 32902353 PMC7717166

[77] Krebs B, Lesica NA, Grothe B. The representation of amplitude modulations in the mammalian auditory midbrain. J Neurophysiol. 2008;100(3):1602–9. doi: 10.1152/jn.90374.2008 18614754

[78] Zheng Y, Escabí MA. Distinct roles for onset and sustained activity in the neuronal code for temporal periodicity and acoustic envelope shape. J Neurosci. 2008;28(52):14230–44. doi: 10.1523/JNEUROSCI.2882-08.2008 19109505 PMC2636849

[79] Johnson JS, Niwa M, O’Connor KN, Sutter ML. Amplitude modulation encoding in the auditory cortex: comparisons between the primary and middle lateral belt regions. J Neurophysiol. 2020;124(6):1706–26. 33026929 10.1152/jn.00171.2020 PMC7814894

[80] Gao X, Wehr M. A coding transformation for temporally structured sounds within auditory cortical neurons. Neuron. 2015;86(1):292–303. doi: 10.1016/j.neuron.2015.03.004 25819614 PMC4393373

[81] Henry KS, Abrams KS, Forst J, Mender MJ, Neilans EG, Idrobo F, et al. Midbrain synchrony to envelope structure supports behavioral sensitivity to single-formant vowel-like sounds in noise. J Assoc Res Otolaryngol. 2017;18(1):165–81. doi: 10.1007/s10162-016-0594-4 27766433 PMC5243265

[82] Henry KS, Neilans EG, Abrams KS, Idrobo F, Carney LH. Neural correlates of behavioral amplitude modulation sensitivity in the budgerigar midbrain. J Neurophysiol. 2016;115(4):1905–16. doi: 10.1152/jn.01003.2015 26843608 PMC4869485

[83] Carney LH, Zilany MSA, Huang NJ, Abrams KS, Idrobo F. Suboptimal use of neural information in a mammalian auditory system. J Neurosci. 2014;34(4):1306–13. doi: 10.1523/JNEUROSCI.3031-13.2014 24453321 PMC3898290

[84] Yao JD, Sanes DH. Temporal encoding is required for categorization, but not discrimination. Cereb Cortex. 2021;31(6):2886–97. doi: 10.1093/cercor/bhaa396 33429423 PMC8107795

[85] Zuo Y, Safaai H, Notaro G, Mazzoni A, Panzeri S, Diamond ME. Complementary contributions of spike timing and spike rate to perceptual decisions in rat S1 and S2 cortex. Curr Biol. 2015;25(3):357–63. doi: 10.1016/j.cub.2014.11.065 25619766

[86] Nelson PC, Carney LH. A phenomenological model of peripheral and central neural responses to amplitude-modulated tones. J Acoust Soc Am. 2004;116(4 Pt 1):2173–86. 15532650 10.1121/1.1784442 PMC1379629

[87] Dicke U, Ewert SD, Dau T, Kollmeier B. A neural circuit transforming temporal periodicity information into a rate-based representation in the mammalian auditory system. J Acoust Soc Am. 2007;121(1):310–26. 17297786 10.1121/1.2400670

[88] Carney LH, McDonough JM. Nonlinear auditory models yield new insights into representations of vowels. Atten Percept Psychophys. 2019;81(4):1034–46. doi: 10.3758/s13414-018-01644-w 30565098 PMC6581637

[89] Hewitt MJ, Meddis R. A computer model of amplitude-modulation sensitivity of single units in the inferior colliculus. J Acoust Soc Am. 1994;95(4):2145–59. doi: 10.1121/1.408676 8201111

[90] Koumura T, Terashima H, Furukawa S. Cascaded tuning to amplitude modulation for natural sound recognition. J Neurosci. 2019;39(28):5517–33. 31092586 10.1523/JNEUROSCI.2914-18.2019 PMC6616280

[91] Masquelier T, Guyonneau R, Thorpe SJ. Spike timing dependent plasticity finds the start of repeating patterns in continuous spike trains. PLoS One. 2008;3(1):e1377. doi: 10.1371/journal.pone.0001377 18167538 PMC2147052

[92] Clarey JC, Paolini AG, Grayden DB, Burkitt AN, Clark GM. Ventral cochlear nucleus coding of voice onset time in naturally spoken syllables. Hear Res. 2004;190(1–2):37–59. 15051129 10.1016/S0378-5955(04)00017-6

[93] Kujawa SG, Liberman MC. Adding insult to injury: cochlear nerve degeneration after “temporary” noise-induced hearing loss. J Neurosci. 2009;29(45):14077–85. 19906956 10.1523/JNEUROSCI.2845-09.2009 PMC2812055

[94] Hockley A, Cassinotti LR, Selesko M, Corfas G, Shore SE. Cochlear synaptopathy impairs suprathreshold tone-in-noise coding in the cochlear nucleus. J Physiol. 2023. 10.1113/JP284452 PMC10374284 37212296

[95] Goodman DFM, Winter IM, Léger AC, de Cheveigné A, Lorenzi C. Modelling firing regularity in the ventral cochlear nucleus: mechanisms, and effects of stimulus level and synaptopathy. Hear Res. 2018;358:98–110. doi: 10.1016/j.heares.2017.09.010 29107413

[96] Bharadwaj HM, Masud S, Mehraei G, Verhulst S, Shinn-Cunningham BG. Individual differences reveal correlates of hidden hearing deficits. J Neurosci. 2015;35(5):2161–72. doi: 10.1523/JNEUROSCI.3915-14.2015 25653371 PMC4402332

[97] Lerud KD, Almonte FV, Kim JC, Large EW. Mode-locking neurodynamics predict human auditory brainstem responses to musical intervals. Hear Res. 2014;308:41–9. doi: 10.1016/j.heares.2013.09.010 24091182

[98] Moore BCJ. An introduction to the psychology of hearing. 4th ed. London: Academic Press; 1997.

[99] Hackney CM, Pick GF. The distribution of spherical cells in the anteroventral cochlear nucleus of the guinea pig. Br J Audiol. 1986;20(3):215–20. doi: 10.3109/03005368609079018 3742109

[100] Osen KK. Cytoarchitecture of the cochlear nuclei in the cat. J Comp Neurol. 1969;136(4):453–84. doi: 10.1002/cne.901360407 5801446

[101] Sayles M, Winter IM. Ambiguous pitch and the temporal representation of inharmonic iterated rippled noise in the ventral cochlear nucleus. J Neurosci. 2008;28(46):11925–38. doi: 10.1523/JNEUROSCI.3137-08.2008 19005058 PMC6671654

[102] Sayles M, Winter IM. Reverberation challenges the temporal representation of the pitch of complex sounds. Neuron. 2008;58(5):789–801. doi: 10.1016/j.neuron.2008.03.029 18549789

[103] Coffey EBJ, Nicol T, White-Schwoch T, Chandrasekaran B, Krizman J, Skoe E, et al. Evolving perspectives on the sources of the frequency-following response. Nat Commun. 2019;10(1):5036. doi: 10.1038/s41467-019-13003-w 31695046 PMC6834633

[104] Rouiller EM, Ryugo DK. Intracellular marking of physiologically characterized cells in the ventral cochlear nucleus of the cat. J Comp Neurol. 1984;225(2):167–86. doi: 10.1002/cne.902250203 6327782

[105] Smith PH, Rhode WS. Structural and functional properties distinguish two types of multipolar cells in the ventral cochlear nucleus. J Comp Neurol. 1989;282(4):595–616. doi: 10.1002/cne.902820410 2723154

[106] Golding NL, Ferragamo MJ, Oertel D. Role of intrinsic conductances underlying responses to transients in octopus cells of the cochlear nucleus. J Neurosci. 1999;19(8):2897–905. doi: 10.1523/JNEUROSCI.19-08-02897.1999 10191307 PMC6782262

[107] Garabedian CE, Jones SR, Merzenich MM, Dale A, Moore CI. Band-pass response properties of rat SI neurons. J Neurophysiol. 2003;90(3):1379–91. doi: 10.1152/jn.01158.2002 12750410

[108] Sanchez-Jimenez A, Panetsos F, Murciano A. Early frequency-dependent information processing and cortical control in the whisker pathway of the rat: electrophysiological study of brainstem nuclei principalis and interpolaris. Neuroscience. 2009;160(1):212–26. doi: 10.1016/j.neuroscience.2009.01.075 19409209

[109] Green DM, Dai HP. Probability of being correct with 1 of M orthogonal signals. Percept Psychophys. 1991;49(1):100–1. doi: 10.3758/bf03211621 2011448

[110] DeCarlo LT. On a signal detection approach to -alternative forced choice with bias, with maximum likelihood and Bayesian approaches to estimation. J Math Psychol. 2012;56(3):196–207. doi: 10.1016/j.jmp.2012.02.004

[111] Green DM, Swets JA. Signal detection theory and psychophysics. New York: Wiley; 1966.

[112] Hofer SB, Mrsic-Flogel TD, Horvath D, Grothe B, Lesica NA. Optimization of population decoding with distance metrics. Neural Netw. 2010;23(6):728–32. doi: 10.1016/j.neunet.2010.04.007 20488662 PMC7618337

[113] Houghton C, Sen K. A new multineuron spike train metric. Neural Comput. 2008;20(6):1495–511. doi: 10.1162/neco.2007.10-06-350 18194108 PMC2782407

[114] Scholes C, Palmer AR, Sumner CJ. Stream segregation in the anesthetized auditory cortex. Hear Res. 2015;328:48–58. 26163899 10.1016/j.heares.2015.07.004 PMC4582803

[115] Peterson AJ, Heil P. Phase locking of auditory nerve fibers: the role of lowpass filtering by hair cells. J Neurosci. 2020;40(24):4700–14. doi: 10.1523/JNEUROSCI.2269-19.2020 32376778 PMC7294794

[116] Kessler D, Carr CE, Kretzberg J, Ashida G. Theoretical relationship between two measures of spike synchrony: correlation index and vector strength. Front Neurosci. 2021;15:761826. doi: 10.3389/fnins.2021.761826 34987357 PMC8721039

[117] Fiore L, Lorenzetti W, Ratti G. Comparing neuronal spike trains with inhomogeneous Poisson distribution: evaluation procedure and experimental application in cases of cyclic activity. J Neurosci Methods. 2005;149(1):7–14. doi: 10.1016/j.jneumeth.2005.03.013 15967509

[118] Joris PX, Louage DH, Cardoen L, van der Heijden M. Correlation index: a new metric to quantify temporal coding. Hear Res. 2006;216–217:19–30. doi: 10.1016/j.heares.2006.03.010 16644160

