# Supplementary material for: Precise spike-timing information in the brainstem is well aligned with the needs of communication and the perception of environmental sounds
Source: PLoS Biol. 2025 Jun 16;23(6):e3003213. doi: 10.1371/journal.pbio.3003213 (PMC12204627; doi:10.1371/journal.pbio.3003213)
Supplement: S1 Text — Fig A. Examples of fitted confusion matrices using the softmax model. A. The example sustained chopper neuron from Fig 2 in the paper. b. The example primary-like neuron from Fig 3 in the paper. c. A primary-like notch neuron which demonstrates a clear where the classifier is systematically bias toward choosing high modulation frequencies. d. An onset-chopper neuron which is less well modeled than 88% of the data. The leftmost panels reproduce the directly calculated confusion matrix. The middle panel shows the predicted confusion matrix from the best fitting values of c′ and B. The rightmost panel shows c′ and B. Dotted lines show the maximum and minimum values derived from 10 separate fits run with different starting parameters, and give some indication of the reliability of the fitting process. The orange line shows the hit rate for comparison. Fig B. Modulation Transfer Functions as a function of sound level when modulation depth is 200%, derived from a spike timing classifier (MTF-c′) or phase-locking (MTF-VS). a. Maximum c′ at the peak of the corresponding modulation transfer function, split by neuron type, and sound level. b. Proportion of MTF-c′ shapes classified as lowpass, split by neuron type and sound level. c. Mean population MTF-c′ for sustained chopper neurons as a function of sound level, frequency-normalized to BMD-c′. d. Mean population MTF-c′ for primary-like neurons as a function of sound level. e. Peak vector strength values and f. proportion of lowpass MTF-VS as a function of neuron type and level. g. Mean population MTF-VS for sustained chopper neurons as a function of sound level, frequency normalized to BMF-VS. h. Mean population MTF-VS for primary-like neurons as a function of sound level. Fig C. Classifier Modulation Transfer Functions (MTF-c′) at shallow modulation depths is 200%. a. Modulation transfer functions in sustained chopper neurons for a modulation depth of 50%. Individual functions where c′ >1 for at least one modulation freque [file pbio.3003213.s001.pdf]

Supplemental Material (S1)

Softmax analysis of confusion matrices as a summary of classifier performance

The *softmax* analysis seeks to summarise the confusion matrix as two separable terms: *c′* which is a metric of classification performance as a function of the presented modulation frequency and *B*, which is a quantification of the bias of the classifier toward individual modulation frequencies independent of which is presented. From c′ and *B*, we can generate a predicted confusion matrix. The RMS error of this prediction (against the actual confusion matrix) was minimised to fit the model. Fig A in S1 Text shows the measured and predicted confusion matrices for the two example neurons (Figs Aa and Ab in S1 Text corresponding to Figs 2 and 3 in the main paper). These demonstrate that it is possible to reproduce the confusion matrices closely. Also shown are the fitted values of *c′* and *b* (right panels). For the example primary-like neuron in particular (Fig Ab in S1 Text, corresponding to Fig 3), it is obvious how *B* varies to model the variations in the probability of choices which are independent of the presented stimulus, which appear as horizontal stripes in the confusion matrix. In comparison, the hit-rates derived from the confusion matrix show a spikey but relatively level function, which would lead one to assume that classification accuracy is fairly uniform across frequency. The *softmax* analysis models this more appropriately as bias plus a clearer low-pass function in *c′*, which is close to zero above 500Hz.

Fig A in S1 Text also shows two further examples. Fig Ac in S1 Text represents a primary-like notch type neuron. In this example there is a clear bias towards choosing high modulation frequencies, and a corresponding increase in *B* with modulation frequencies. This is independent of the stimulus, and therefore we can regard it as a nuisance parameter. The hit rates at high frequencies are slightly inflated by this, whereas the *softmax* analysis allows us to factor it out. The final example (Fig Ad in S1 Text) is an onset chopper neuron, which is presented as an example of a poorer fit than was usual. This shows a bias towards modulation frequencies close to 1000Hz, which is well accounted for by *B*, and at a glance it models the confusion matrix well. In comparison, the hit-rates are inflated in this frequency region. However, there are some errors around the main diagonal in the confusion matrix, which indicate that errors often involved choice of nearby modulation frequencies. These are not captured by the model, which assumes that the probability of *incorrect* choices is unaffected by the stimulus. A more complex model could explicitly model these local errors, but this was not considered warranted. The RMS error is larger than 88% of the fits across the dataset, at 0.36 (units of probability). Thus, for the majority of the data the analysis we employ provides a better summary of classification performance than this example.

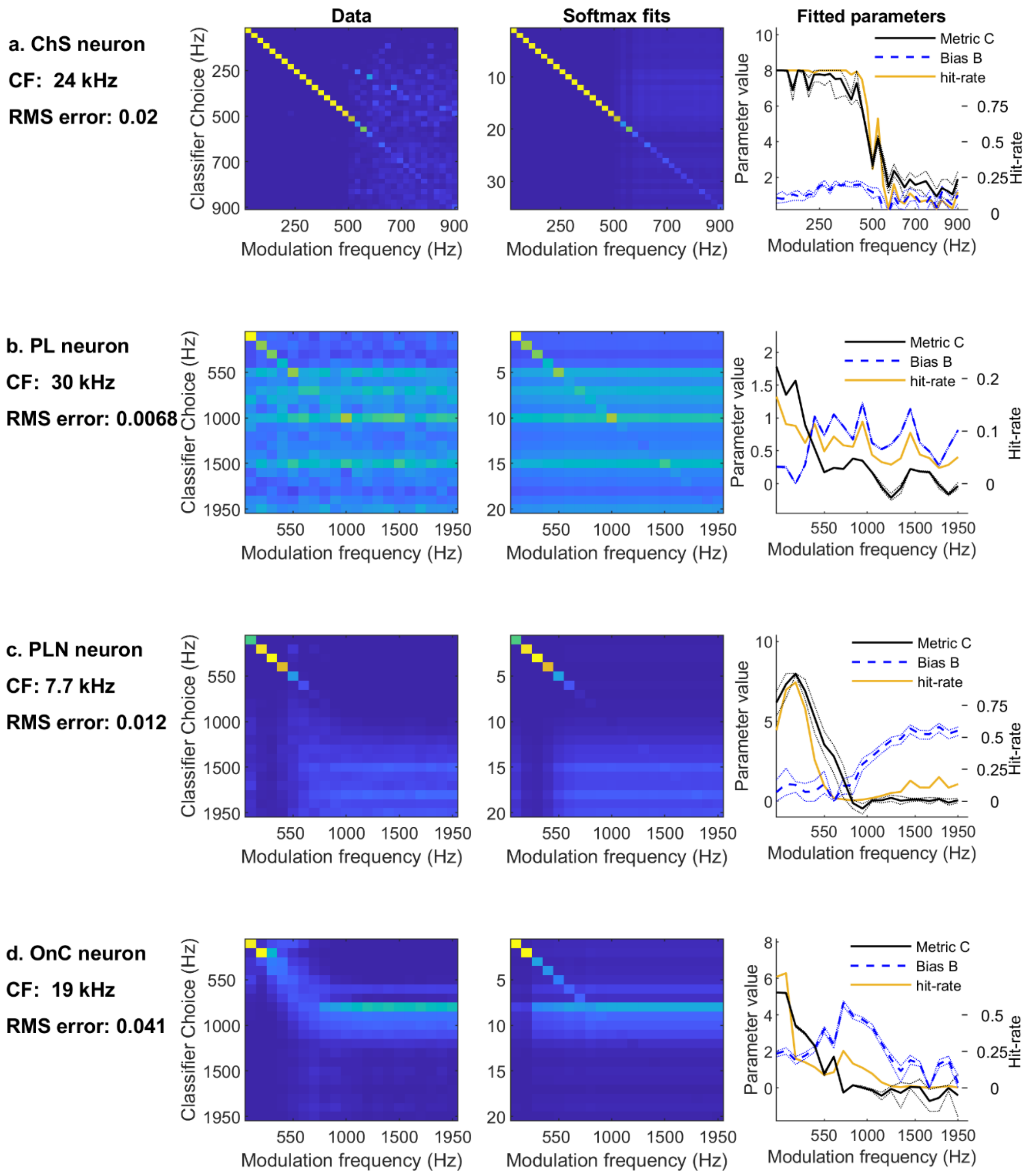

Fig A. Examples of fitted confusion matrices using the *softmax* model. a. The example sustained chopper neuron from Fig 2 in the paper. **b.** The example primary-like neuron from Fig 3 in the paper. c. A primary-like notch neuron which demonstrates a clear where the classifier is systematically bias towards choosing high modulation frequencies. **d.** An onset-chopper neuron which is less well modelled than 88% of the data. The leftmost panels reproduce the directly calculated confusion matrix. The middle panel shows the predicted confusion matrix from the best fitting values of *c′* and *B*. The rightmost panel shows *c′* and *B*. Dotted lines show the maximum and minimum values derived from 10 separate fits run with different starting parameters, and give some indication of the reliability of the fitting process. The orange line shows the hit-rate for comparison.

The influence of modulation depth and modulation frequency differences on discrimination

While we focus on envelope coding of fully modulated tones in the main text (100%; where the amplitude in the minima is zero), the dataset (1, 2) also includes responses to over-modulated (200%)(2) and shallow modulation depths (20%, 50%). The data at modulation depths other than 100% reveal that our main conclusions hold regardless of modulation depth.

Fig B in S1 Text is an alternative version of Fig 7 in the main paper, which shows the same results for a modulation depth of 200%. Comparing Fig B in S1 Text with Fig 7, all the same trends can be observed. In fact, an even larger proportion of discrimination functions are lowpass in shape.

There are fewer data available at shallow modulation depths. Fig C in S1 Text shows the classification functions of all available neurons, with the bulk coming from sustained choppers (15 ChS, 3 PLN, 3 On, 2 PL and 1 PBU) spread across the two modulation depths. Classification performance at shallow modulation depths is lower than observed for 100% modulation in all neuron types. In those responses where *c*′>1 for at least one modulation frequency, most of the functions were lowpass in shape. The small and uneven sampling of different neuron types makes it challenging to draw meaningful conclusions about differences between them. Note that responses to 20% modulation depths were not included in the statistical regression analysis.

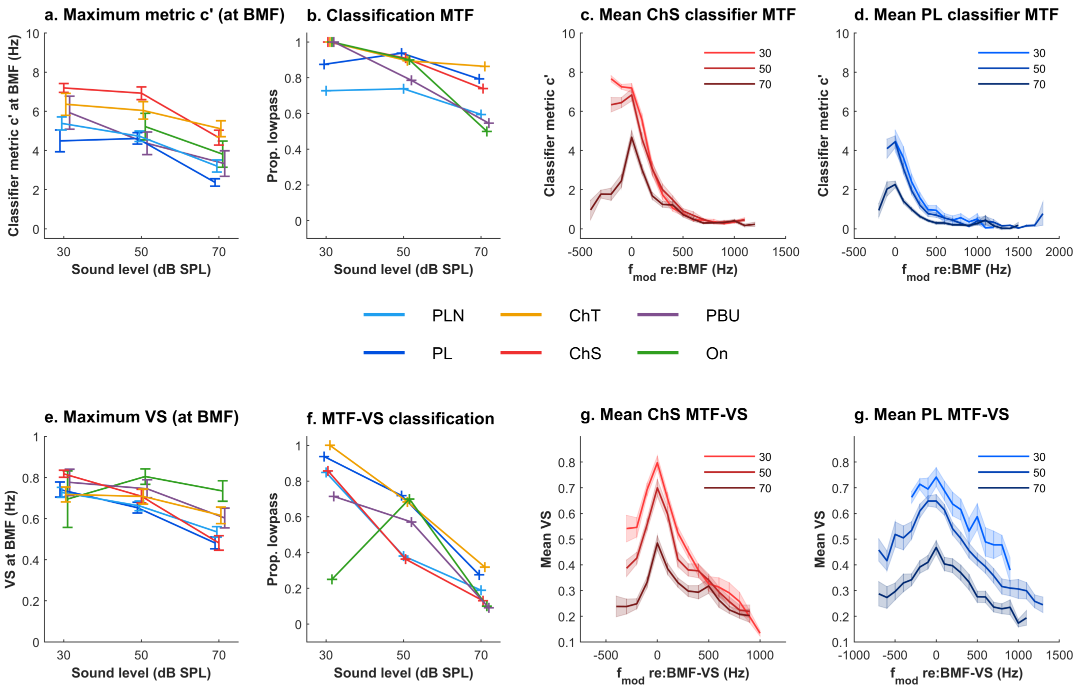

Fig B. Modulation Transfer Functions as a function of sound level when modulation depth is 200%, derived from a spike timing classifier (MTF-*c*′) or phase-locking (MTF-VS). a. Maximum *c*′ at the peak of the corresponding modulation transfer function, split by neuron type, and sound level. **b.** Proportion of MTF-*c*′ shapes classified as lowpass, split by neuron type and sound level. **c.** Mean population MTF-*c*′ for sustained chopper neurons as a function of sound level, frequency-normalized to BMD-*c*′. **d.** Mean population MTF-*c*′ for primarylike neurons as a function of sound level. **e.** Peak vector strength values and **f**. proportion of lowpass MTF-VS as a function of neuron type and level. **g.** Mean population MTF-VS for sustained chopper neurons as a function of sound level, frequency normalized to BMF-VS. **h.** Mean population MTF-VS for primarylike neurons as a function of sound level.

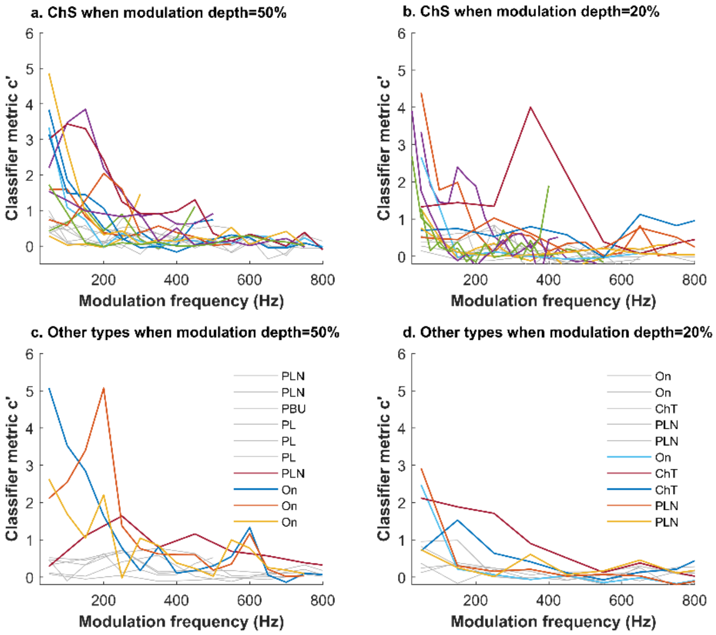

Fig C. Classifier Modulation Transfer Functions (MTF-*c′*) at shallow modulation depths is 200%. a. Modulation transfer functions in sustained chopper neurons for a modulation depth of 50%. Individual functions where *c′*>1 for at least one modulation frequency are shown as colored lines (n=12). Functions which do not exceed *c′*=1 are shown in grey (n=7). b. Modulation transfer functions in sustained chopper neurons for a modulation depth of 20%. c. Modulation transfer functions in all other neuron types for a modulation depth of 50%. d. Modulation transfer functions in all other neuron types for a modulation depth of 20%.

We were also able to examine the generality of our results for smaller differences in modulation frequency, for a small sample of neurons (7 ChS, 1 PBU). Fig D in S1 Text shows the modulation classification functions for sets of stimuli where the difference between modulation frequencies was 25Hz or less. MTF-*c′* functions were again low-pass in shape, and overall discrimination dropped with increasing sound level. It is also noteworthy that overall classifier metric (*c′*) values in sustained choppers could nevertheless be similar (*c′*>5) to those seen in the dataset generally, where modulation frequencies typically differed by 100Hz (c.f. Fig 7 in the main paper).

The dataset contained one neuron, a transient chopper, where the modulation frequencies differed by only 5Hz (Fig Db in S1 Text), and were limited to modulation frequencies below 50Hz. In this neuron, modulation frequency identification was strong (*c′*>5) at the lowest sound level, functions were fairly flat, and overall classification decreased with low sound levels.

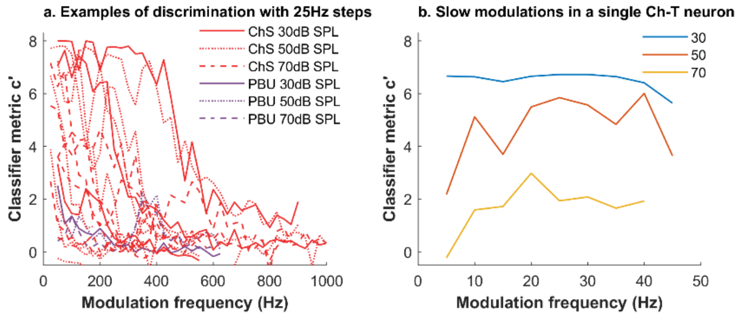

Fig D. Classifier Modulation Transfer Functions (MTF-*c'*) for small differences in modulation frequency. a. Modulation transfer functions in 7 sustained chopper neurons and one pause/build-up neuron for a modulation frequency steps of 25Hz. Line style represents sound level. Different lines with the same style are different example neurons. **b.** Modulation transfer functions in a single transient chopper-neuron at very low modulation frequencies (<50Hz) and very small frequency steps (5Hz) at several sound levels.

The Z_{ISI} statistic

Fig E in S1 Text shows how the Z_{ISI} statistic is dependent on modulation frequency. It generally decreases with modulation frequency, but the relationship between neuron types remains largely unchanged. In sustained chopper and pause buildup neurons, the dependence on modulation frequency is less than in other neuron types. It should be noted that we do not compute Z_{ISI} if VS is not significant (Rayleigh>13.8), since phase-shuffling makes no sense without some dependence on phase, so these functions become biased toward a smaller number of neurons as modulation frequency increases. Fig Eb in S1 Text shows Z_{ISI} for all neuron types, as the mean value of Z_{ISI} for all modulation frequencies < 1kHz for each dataset, showing a similar dependence on neuron type as for f_{mod}~150. Regardless of the way the statistic is summarised within a neuron, it demonstrates robust differences across neuron types.

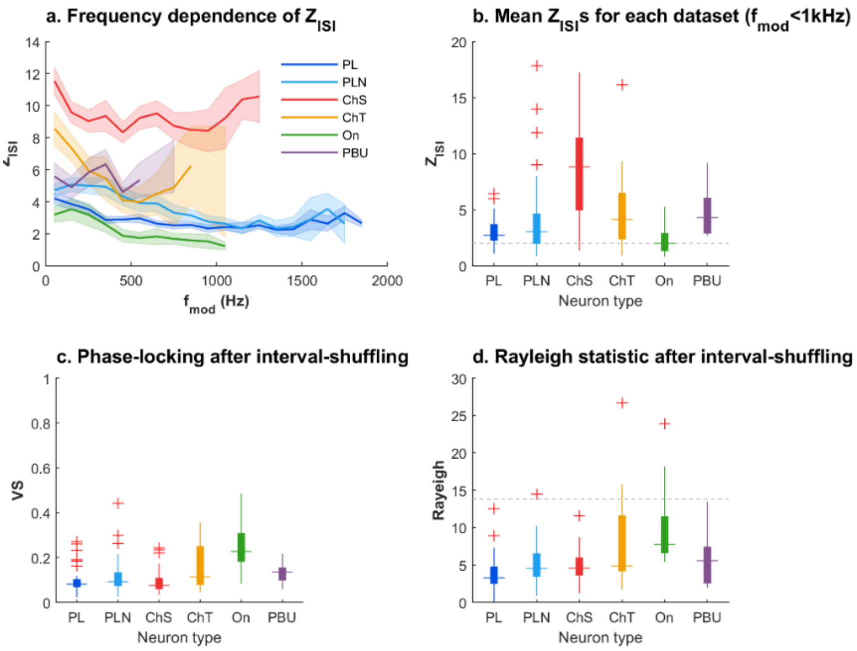

Fig E. Details of the behaviour of the Z_{ISI} statistic. a. The frequency dependence of Z_{ISI} for each neuron type. Shaded areas show standard error of the mean. **b.** Mean values of Z_{ISI} from each dataset, calculated as the average of all values for f_{mod}<1kHz. Dashed line indicates a value of 2 (units are standard deviation) which was proposed by Laudanski et al. (3) as one of the criteria for mode-locking. **c.** Mean values of Vector Strength for each dataset (mean for f_{mod}<1kHz), following the interval shuffling proposed as the second criteria for mode-locking. **d.** Rayleigh criterion values following interval shuffling. Laudanski et al. proposed phase locking should be nonsignificant following interval shuffling if responses were mode-locked. Dashed line indicates a Rayleigh value of 13.8 (below this corresponding to p>0.001).

Laudanski et al.'s previous "test" for mode-locking

The Z_{ISI} statistic was originally proposed as part of a formal "test" for mode-locking (3). The test was motivated by our observations in a small sample of Chopper neurons and a larger set of Onset neurons, recorded in the ventral cochlear nucleus of guinea-pigs. The dataset in the current paper, with a sample of many of the different neuron types in this region, showed that this test does not discriminate across neuron types, and therefore had little explanatory power. The Z_{ISI} statistic itself does however remain a powerful analysis tool, which is why we use it here.

The first part of the proposed formal test was that Z_{ISI} should be >2. This would indicate the effect of phase-shuffling on the interval distribution was two standard deviations greater than the variability of the unshuffled interval distribution (the method itself is described in the main paper). According to Fig Ea and Eb in S1 Text, most neurons of all types pass this test, with the exception of onset neurons. Around half of onset neurons pass the test, which is consistent with Laudanski et al.(3). Thus, Z_{ISI} is a useful statistic which discriminates between types of neurons, but the proposed threshold is too low. This is likely to reflect that all neurons possess a short refractoriness, which introduced sufficient independence between the phase and interval statistics to be detected by Z_{ISI}.

The second part of the formal test for mode-locking proposed by Laudanski et al.(3) was that shuffling the interspike intervals (whereas Z_{ISI} involves shuffling spike times between phases) should destroy phase-locking, and result in a non-significant Rayleigh statistic. Fig Dc and Dd in S1 Text show the VS and Rayleigh statistics following interval shuffling described in (3), split by type and averaged within each data set for f_{mod}<1kHz. It shows that VS is very low following interval shuffling (c), and very few neurons show significant phase locking (Rayleigh statistic >13.8 which corresponds to p<0.001). Thus, both components of the previously proposed test of mode-locking are weak and would lead to the conclusion that almost all neurons showed mode-locking. This contrasts with our new proposed explicit test based on the SAC functions.

It would of course be possible to create a better binary test by choosing new decision criteria, or using SAC-based statistics. In the current study we took a different approach since a binary decision as to whether or not a neuron showed mode-locking was not appropriate as a predictor in our statistical regression models.

The use of statistics at f_{mod}~150Hz in statistical regression models.

In Fig 9a of the main paper we compare how different spike train statistics predict classifier performance across the entire data. The choice of statistics shown was limited, for clarity and for several additional principled reasons.

A key decision was to take from each dataset (a single "dataset" being the responses to a set of modulation frequencies, from a particular neuron at a given sound level and modulation depth) a single value for each statistic. Importantly for comparison between models, this meant that the statistical regression models could only predict changes in the overall classifier performance between neurons. They could not predict any differences in the MTF-shape. Thus, any differences in how well different models fit the data can be safely interpreted as differences between neurons. It also meant that the theoretical maximum for the model fit was known (~79% of variance accounted for). A further advantage of this method was that the statistic used was not drawn from the exact same spike trains as it predicted (except at one modulation frequency), so was unlikely to reflect an overfit to the data.

For completeness, Fig F in S1 Text shows an expanded version of Fig 9a. It shows the model performance when each statistic was derived separately at each modulation frequency (purple bars). These values show most of the same trends, and still support our conclusions, though they vary in several respects. In some cases (VS, SAC peaks, reliability) the prediction is improved by 0.05% or greater. This is what we would expect, since it is these statistics are all to varying degrees indexing information which should be useful for identifying modulation frequency. An increased predictive power could indicate that the variation in the statistics with modulation frequency mirror those of *c′* for individual datasets. However, for the remaining statistics, the predictive power is relatively unchanged. Change is less likely in measures that are more highly correlated across modulation frequency, and less closely related to variations in frequency dependence of *c′*.

Fig F in S1 Text also includes several additional statistics not shown in the main results. Correlation index (CI) is the height of the SAC at zero lag, which is correlated to the peak-to-trough height of the peak at zero (R^2 = 0.29). We also show the result of number of significant SAC peaks as a predictor. For the f_{mod}~150Hz statistic, this offers similar predictive power to the peak-to-trough height at the modulation period, but is less powerful when taken from each modulation frequency. SAC peak-to-trough statistics are analysed further below.

Neural fluctuation is a measure of the sum of the firing rate gradient across the response to a given stimulus (4). This statistic is expected to grow monotonically with VS, but would be larger still for responses where there were more fluctuations per period, as would be the case for mode-locking. This a strong predictor and adds further support to the hypothesis any reliable temporal features are likely to be useful for discrimination. We did not include this in the main results because it does not separate out the contributions of phase-locking and mode-locking and thus we did not feel it had much explanatory power. Figu F in S1 Text also shows various combinations of these four most predictive statistics and again supporting that the various correlated variables are of similar explanatory value (pair-wise correlations are presented below).

The choice of taking each statistic at f_{mod}~150Hz, as opposed to another modulation frequency, or some combination of modulation frequencies (e.g. averaging below 500Hz or 1kHz), was not critical to the conclusions. All statistics displayed a drop in their value and their range with increasing modulation frequency, so a high modulation frequency would yield a weaker measure with less reliable differences (e.g. see Fig F in S1 Text for Z_{ISI}) between both neuron types and individual datasets. However, since all the statistics were strongly correlated with the values at neighbouring modulation frequencies, any value or combination of values from low modulation frequencies would yield similar results (Pearson correlations between values at adjacent modulation frequences for f_{mod}<1kHz are 0.89 or larger for Z_{ISI}, Reliability, and the amplitude of the 1st SAC peak; for neural fluctuation the value is 0.86. For the amplitude of the 2nd SAC peak, which in non-zero only in a minority of neurons and only at low frequencies, the value is 0.42).

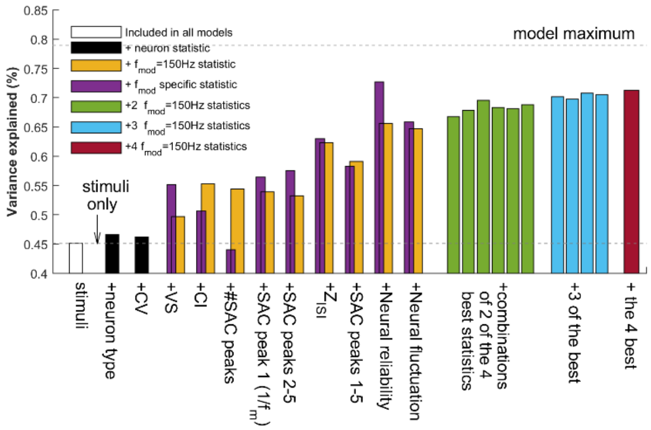

Fig F. Further comparisons of how different statistics predict classification performance. In most cases, the statistics are drawn from f_{mod}~150Hz and applied across all modulation frequencies within a dataset, and the stated number of parameters is added to a model with stimulus-only parameters. Purple bars show the predictive power when the statistics are instead drawn from the specific modulation frequencies. Note that the theoretical maximum does not apply to the purple bars, since the predictions can vary in frequency dependence (transfer function shape) between datasets. The underlying data for this figure is contained in S4 Data.

The number and frequency of peaks in the SAC function

The peak-picking algorithm which we apply to the SAC function seeks to recursively divide the function into a series of maxima and minima. At each division it is determined whether the new peak-to-trough amplitude is significantly higher than that expected from a spike-train with the same number of spikes but no temporal structure (see Methods). The algorithm arrives at a final list of significant peaks and outputs the location and peak-to-trough amplitude of each peak. If a neuron displays robust phase-locking it is expected to exhibit a peak at zero-lag, and successively smaller peaks at the delays associated with multiples of the modulation period, but no additional peaks. Since the SAC function is symmetrical about the zero-lag, and repetitive, we only analyse delays from -0.1 modulation periods (negative of the zero-lag) to 1.1 periods positive.

Fig G in S1 Text shows the occurrence of SACs with different numbers of peaks in different neuron types. In sustained chopper neurons peaks in the SAC function at delays less than the modulation period were most common at low modulation frequencies. This trend can be seen but is less reliable in pause/build-up neurons, whilst at in all other neurons the trend is if anything reversed, with more sub-modulation period peaks occurring at high modulation frequencies. However, the peaks observed at high modulation frequencies were small, occurred at very short intervals, and mainly reflect the limitations of the peak-picking algorithm which becomes problematic when there are no larger peaks in the SAC. For example, for there to be 7 peaks in the SAC function at a modulation period of 1.1ms (950Hz), there would need to be intervals occurring at multiples of 0.15ms. Small ripples can be seen in the data at high modulation frequencies but we did not investigate their origin. Evidence of *robust* interspike intervals at less than the modulation period, which are indicative of mode-locking and contribute to modulation discrimination, were limited to sustained chopper and pause/build-up neuron types at low modulation frequencies.

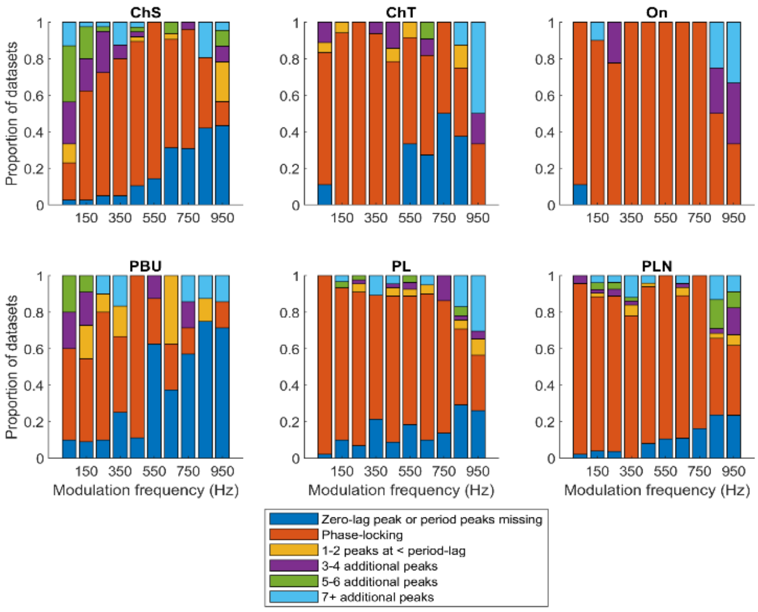

Fig G: Frequency dependence of the number of significant SAC peaks for each neuron type. The underlying data for this figure is contained in S5 Data.

The choice of SAC peaks in statistical regression models

Fig H in S1 Text shows how the different peaks in the SAC at f_{mod}~150Hz are related to envelope classification performance, through incremental additions and removal of individual peaks from the statistical regression models. Peaks at zero lag and the modulation periods are highly correlated (R^2=0.81) and combining them adds little to the predictive power of models, but it does not matter which one is used as a predictor. However, adding the additional peaks which occur at less than the modulation period incrementally adds to the model prediction. These observations justify the omission of the zero-lag peak and the pragmatic lumping together of the additional peaks in the main results. We did not quantify the predictive value of more than peaks 0-5.

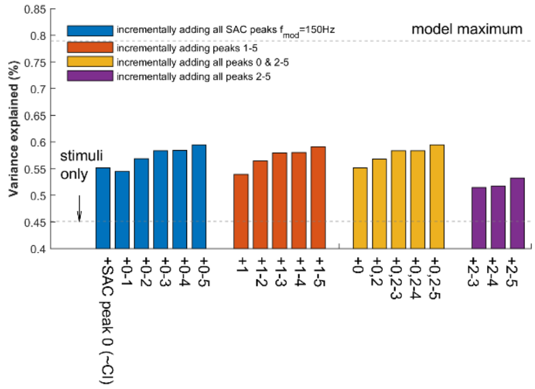

Fig H. How different SAC peaks contribute to predictions of classification performance. In all cases, the statistic is drawn from f_{mod}~150Hz and applied across all modulation frequencies within a dataset, and the stated number of parameters is added to a model with stimulus-only parameters. The underlying data for this figure is contained in S6 Data.

Alternative measures of classifier performance

The *softmax* derived metric, *c'*, was used to summarise classifier performance because it could account for much of the variation in the calculated confusion matrix, including the influence of the number of modulation frequencies at which each neuron was tested (see Methods). Nevertheless our results and conclusions are robust to a range of other metrics.

Fig I in S1 Text reproduces relevant panels from Figs 4, 5, 8 and 9 using hit-rate (also known as recall rate), which is the proportion of correct choices when a given modulation frequency is presented, $p(f_{mod}$ *chosen* $|$ f_{mod} *presented)*. Hit-rate based transfer functions are low-pass (Fig Ia in S1 Text, for the same examples as in Fig 4), and in sustained and transient chopper neurons functions are more uniform in their low-pass shape than other measures, with perfect hit-rates being common at low-modulation frequencies at low sound levels. Note that hit-rate does not account for false-alarms, and therefore provides an incomplete and in this instance optimistic description of classifier performance. The majority of transfer functions are low-pass in all neuron types, with most BMFs being close to the lowest frequencies tested (Fig Ib and c in S1 Text). Average MTFs across all neuron types display a characteristic low-pass shape which differs less between neuron types than do overall differences in hit-rate. Statistical regression models can account for a smaller proportion of the variation in hit-rate compared with *softmax* analysis (theoretical maximum is 73% of variance vs 79%; Fig If in S1 Text), yet the differences in predictive power across different models is qualitatively unchanged (Fig If in S1 Text), offering further support for the conclusions based on the *softmax* analysis. Classification of modulation frequency based on clusters of neurons again displays increased level robustness compared with single neuron performance and the same differences in level-robustness across neuron type (Fig Ig in S1 Text).

We also repeated the analysis for a 3rd measure, F1 score, which is the harmonic mean of the hit-rate (recall), and *precision*:

F1-score = 2 x precision x hit-rate/(precision + hit-rate)

Where precision is the proportion of times a modulation frequency is correctly chosen compared with all the times that modulation frequency is chosen (correctly or incorrectly):

Precision = number of true-positives/(number of true positives + number of false positives)

This measure has the benefit of taking account of false-positives, whilst being simpler than the *softmax* function in not requiring fitting or making specific assumptions about the structure of the confusion matrix. It does not however account for the number of modulation frequencies tested, and like hit-rate it is somewhat subject to ceiling and floor effects.

Fig J in S1 Text reproduces the same set of figure panels from the paper as in Fig I in S1 Text but with F1-score as the measure of classifier performance. Analysis based on F1-score again supports our conclusions: that classification of modulation frequency is a predominantly low-pass characteristic, particularly in sustained chopper neurons which encode envelope best, that good envelope classification is best described by reliable, non-Poisson like spike-timing, and that classification becomes more robust to changes with sound level in clusters of sustained chopper neurons.

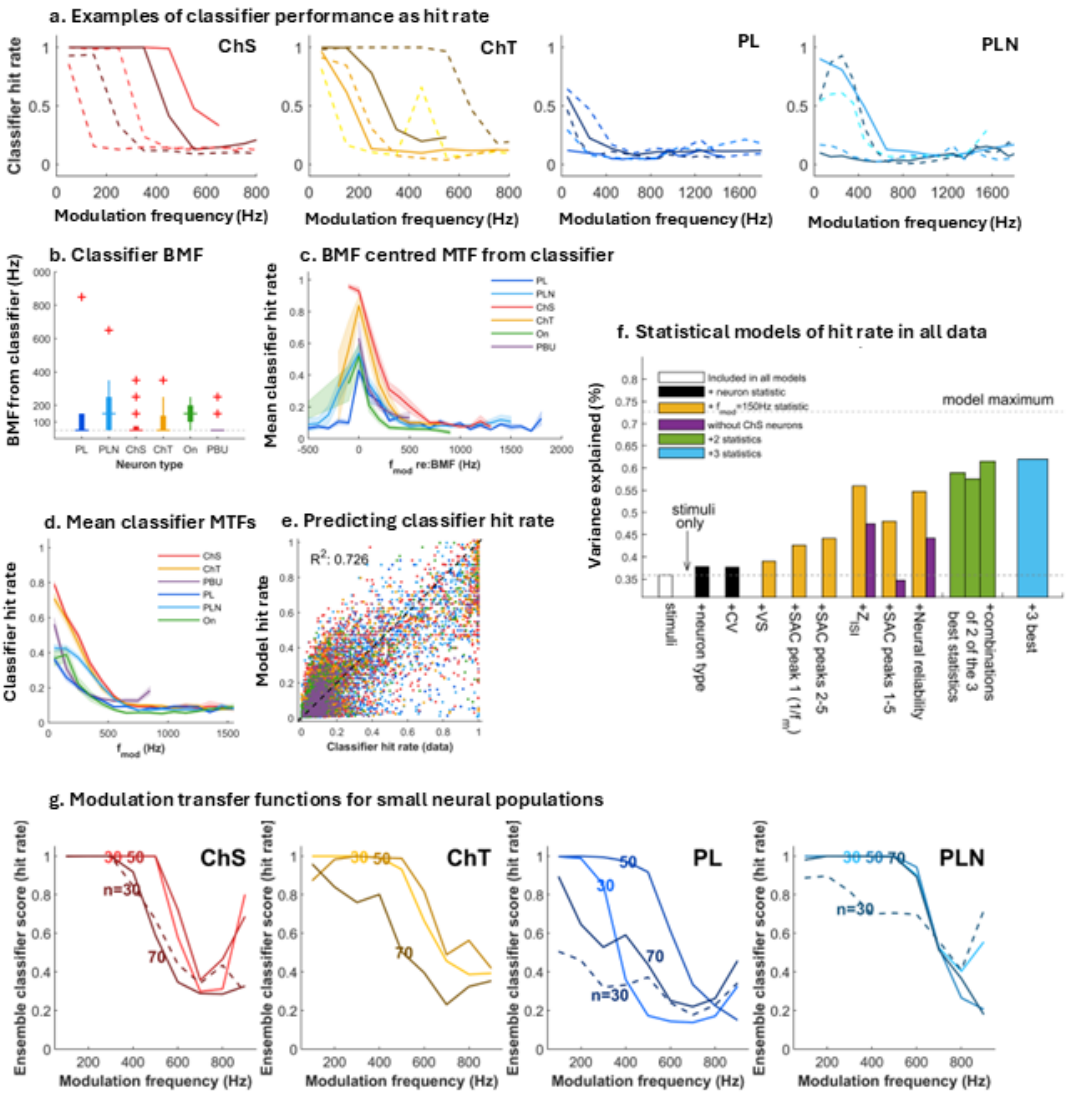

Fig I. Reproducing the main findings using classifier hit-rate. a. Examples of classifier performance as hit rate, for the exact same sets of neurons as in Fig 4. **b.** The range best modulation frequencies when classification is expressed as hit rate, for each neuron type at low-level sound levels. This reproduces Fig 6a. **c.** Mean of modulation transfer functions relative to hit rate BMF, at low sound levels. This reproduces Fig 6b. **d.** Mean of modulation transfer functions for each neuron type as a function of absolute modulation frequency, reproducing Fig 5a. **e.** The best possible prediction of hit rate when the transfer function is modelled as a single function which can only differ by a scale factor between neurons, sound levels and modulation depth. **f.** Reproduction of the statistical regression models from Fig 9a, when classifier performance is expressed as hit rate. **g.** Modulation transfer functions for small populations of neurons when hit rate is the measure of classifier performance. The underlying data for this figure is contained in S7 Data.

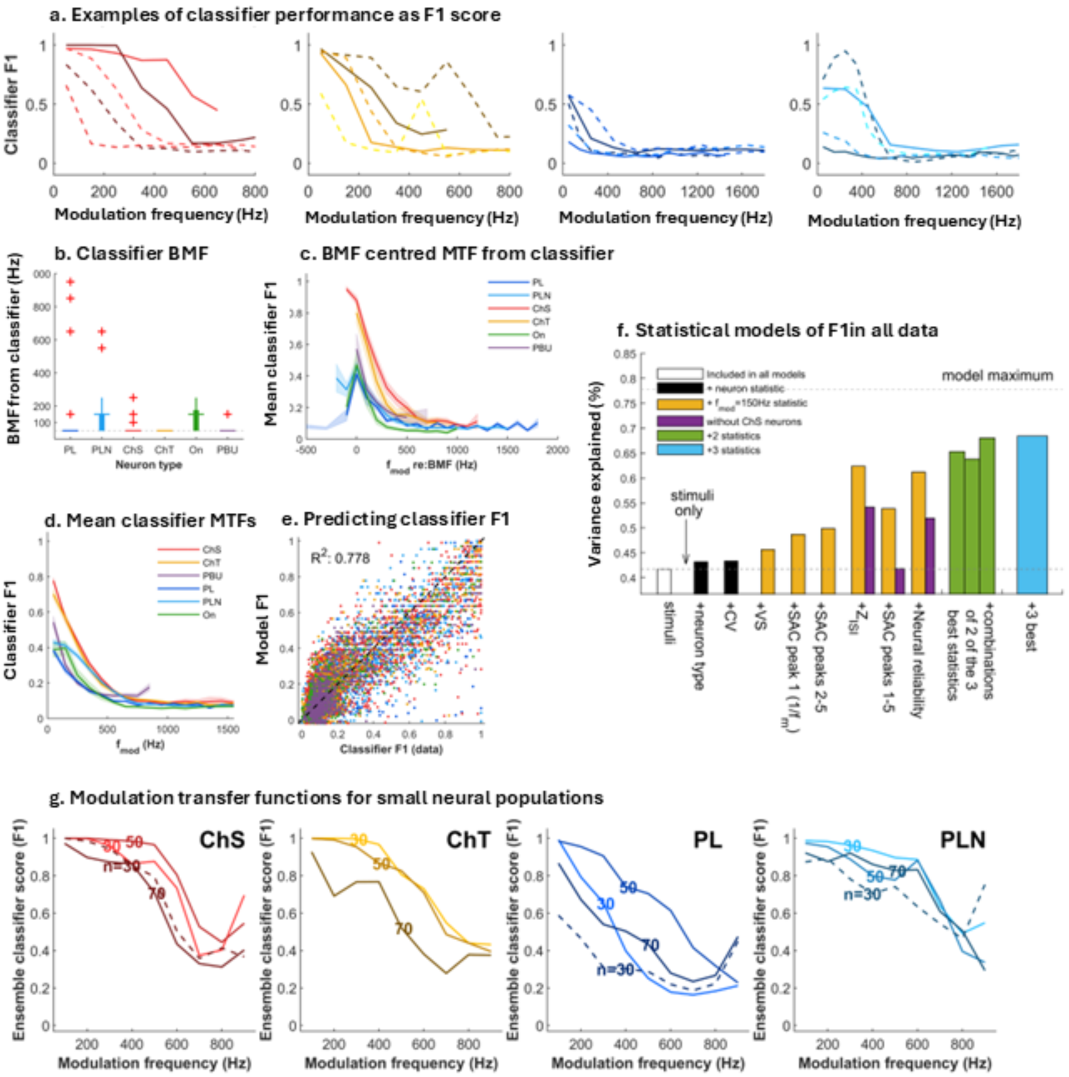

Fig J. Reproducing the main findings using classifier F1-score. a. Examples of classifier performance as F1-score, for the exact same sets of neurons as in Fig 4. **b.** The range best modulation frequencies when classification is expressed as F1 score, for each neuron type at low-level sound levels. This reproduces Fig 6a. **c.** Mean of modulation transfer functions relative to F1-score BMF, at low sound levels. This reproduces Fig 6b. **d.** Mean of F1 modulation transfer functions for each neuron type as a function of absolute modulation frequency, reproducing Fig 5a. **e.** The best possible prediction of F1-score when the transfer function is modelled as a single function which can only differ by a scale factor between neurons, sound levels and modulation depth. **f.** Reproduction of the statistical regression models from Fig 9a, when classifier performance is expressed as F1-score. **g.** Modulation transfer functions for small populations of neurons when F1-score is the measure of classifier performance. The underlying data for this figure is contained in S8 Data.

Correlations between predictors

The various predictors we tested in the statistical regression models were strongly correlated with each other. Thus, although they quantified distinct conceptual aspects of the spike trains, these were in practice not separable. Fig K in S1 Text shows the pair-wise correlations between all the high-performing statistics, and classification performance (*c′*), for f_{mod}~150Hz. This demonstrates that there is probably no way to separate the roles of these different aspects of the spike train.

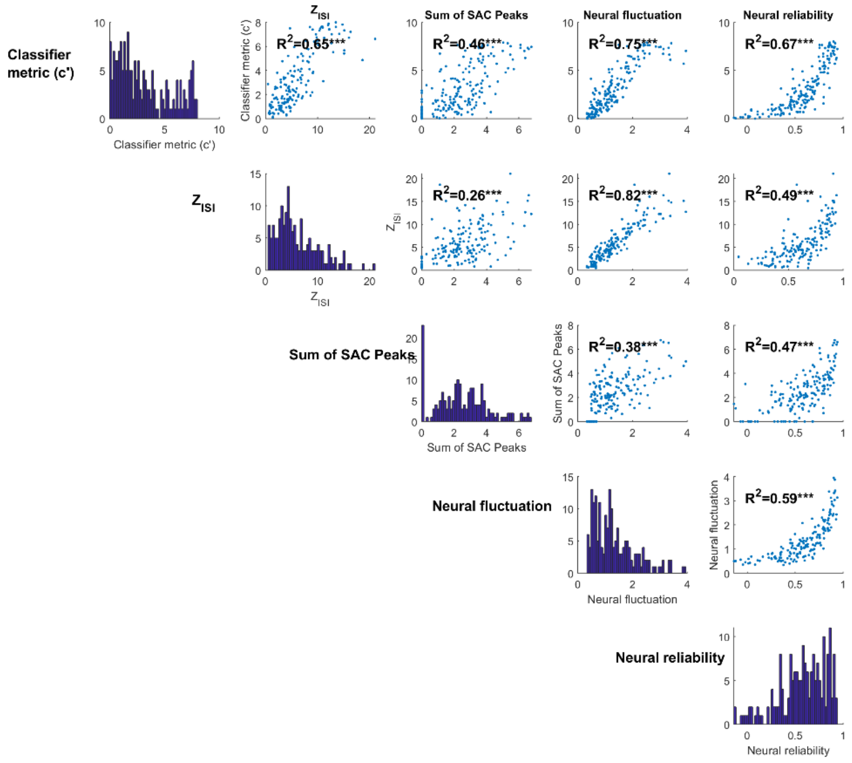

Fig K. Pairwise correlations between the various predictors in the statistical regression. The statistics apply to responses for f_{mod}~150Hz. To reduce the total number of plots, the SAC peaks are shown as a simple unweighted sum.

The temporal resolution of spike-timing to the envelope

In our main analysis we ran the classifier at a range of time windows for all datasets, and for each dataset we picked the classifier which yielded the highest mean *c′* across modulation frequency. The "best-classifier" resolutions chosen are shown in Fig L in S1 Text, split by neuron type. These results concord well with what we know about the membrane time constants in the cells associated with the different response types. The majority of Primary-Like and Primary-Like Notch neurons, associated with bushy cells which show little temporal integration, yield the best performance at the shortest time window (τ = 1ms). Sustained-Chopper neurons, which are associated with T-stellate cells which show temporal integration, show a tendency to perform best at slightly longer time windows (τ ~2ms).

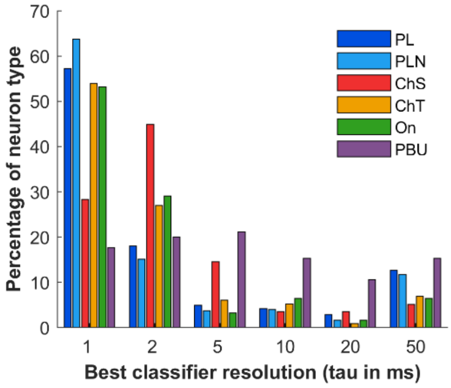

Fig L. The classifier resolutions which yield the best performance, split by neuron type. The underlying data for this figure is contained in S9 Data.

To evaluate how important the precision of spike-timing is to envelope classification, we re-ran some of our analyses using classifiers of fixed temporal resolution of 10ms. This is a lower temporal resolution than optimal in the 80% of neurons. Fig M in S1 Text reproduces the relevant panels from Fig 6 of the main paper, alongside the equivalent analysis for τ = 10ms. This demonstrates that there is useful information at the millisecond timescale, since the functions are more severely lowpass at the lower temporal resolution. However, a low temporal resolution does not qualitatively change our evaluation of the neurons' behaviour.

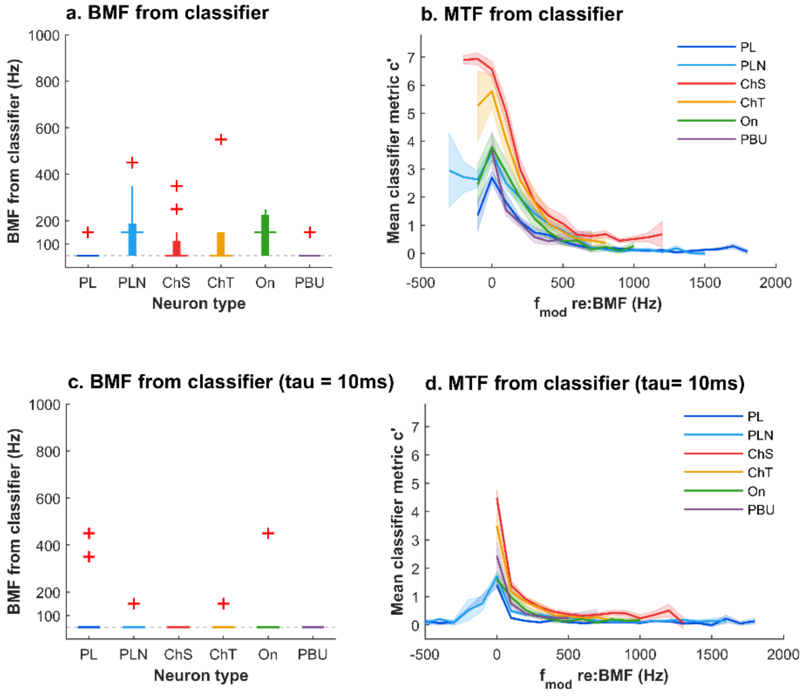

Fig M. Partial reproduction of Fig 6 in the main paper showing the impact of a reduced temporal resolution in the classifier. Upper panels (a) and (b) show results reproduce these panels as they appear in the main paper, where the classifier resolution was chosen to maximise the performance of each MTF. Lower panels (c) and (d) show the equivalent analysis for a fixed classifier resolution of τ =10 ms. The underlying data for this figure is contained in S10 Data.

In Fig N in S1 Text we repeat the same exercise with Fig 7 from the main paper. This again shows that the shapes of the classification functions are unchanged, and they are also similarly affected by sound level.

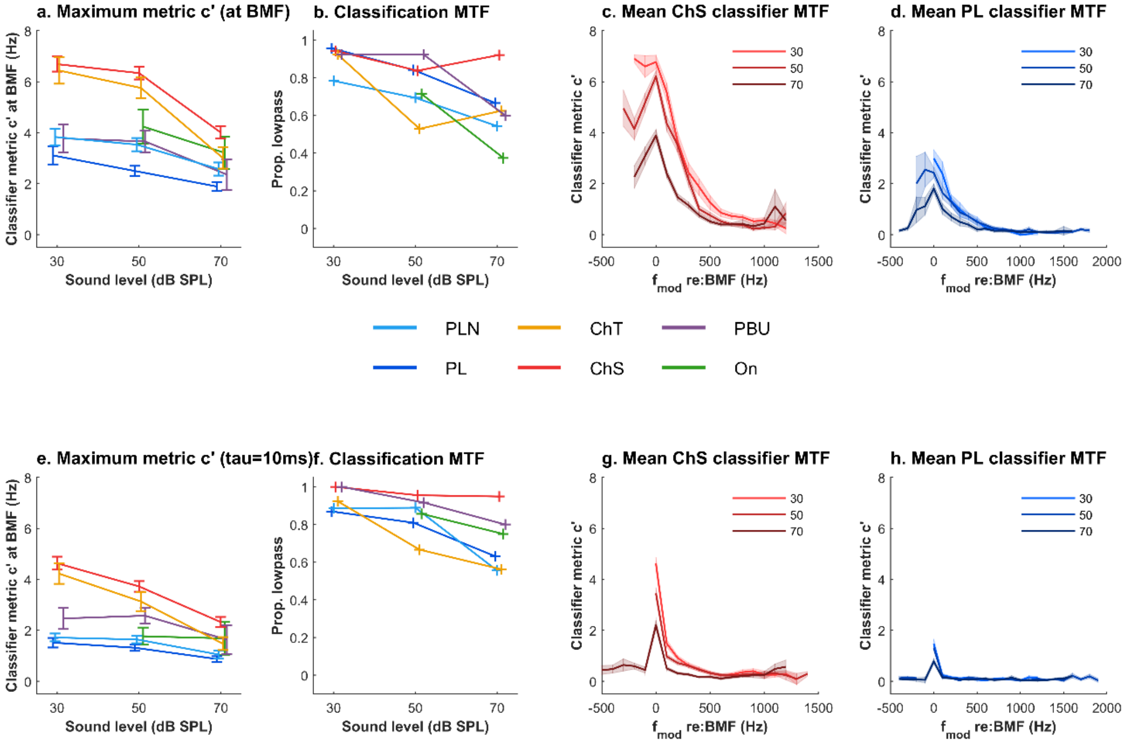

Fig N. Reproduction of Fig 7 in the main paper showing the impact of a reduced temporal resolution in the classifier. Upper panels (a)-(d) reproduce these panels as they appear in the submitted manuscript, where the classifier resolution was chosen to maximise the performance of each MTF. Lower panels (e)-(h) show the same analysis for a fixed classifier resolution of τ =10 ms.

References

1. Rhode WS, Greenberg S. Encoding of amplitude modulation in the cochlear nucleus of the cat. J Neurophysiol. 1994;71(5):1797-825.
2. Rhode WS. Temporal coding of 200% amplitude modulated signals in the ventral cochlear nucleus of cat. Hear Res. 1994;77(1-2):43-68.
3. Laudanski J, Coombes S, Palmer AR, Sumner CJ. Mode-locked spike trains in responses of ventral cochlear nucleus chopper and onset neurons to periodic stimuli. J Neurophysiol. 2010;103(3):1226-37.
4. Gai Y, Carney LH. Temporal Measures and Neural Strategies for Detection of Tones in Noise Based on Responses in Anteroventral Cochlear Nucleus. Journal of Neurophysiology. 2006;96(5):2451-64.
